# Supplementary material for: Non-Target Metabolomics Reveals Changes in Metabolite Profiles in Distant Hybrid Incompatibility Between Paeonia sect. Moutan and P. lactiflora
Source: Plants (Basel). 2025 May 3;14(9):1381. doi: 10.3390/plants14091381 (PMC12073477; doi:10.3390/plants14091381)
Supplement: Supplementary file 1 [file plants-14-01381-s001.zip › plants-3557173-supplementary.pdf]

Supplementary Material

Non-target metabolomics reveals changes in metabolite profiles in distant hybrid incompatibility between *Paeonia* sect. *Moutan and P. lactiflora*

Wenqing Jia[1,*], Yingyue Yu [1], Zhaorong Mi [1], Yan Zhang [1], Guodong Zhao [2], Yingzi Guo [3], Zheng Wang [3], Erqiang Wang [4] and Songlin He [3,*]

1 School of Horticulture and Landscape Architecture, Henan Institute of Science and Technology, Xinxiang 453003, China; mint_19@163.com (Y.Z.); mizr@hist.edu.cn (Z.M.); yuyingyue0923@163.com (Y.Y.)
[2] Luoyang National Peony Gene Bank, Luoyang 471011, China; zhaoguodong2025@163.com (G.Z.)
[3] College of Landscape Architecture and Art, Henan Agricultural University, Zhengzhou 450002, China; guoyz2017@126.com (Y.G.); wzhengt@163.com (Z.W.)
[4] Luoyang Academy of Agricultural and Forestry Sciences, Luoyang 471027, China; 1078070473@qq.com (E.W.)
* Correspondence: jiawq2022@hist.edu.cn (W.J.), Tel.: +86-373-369683 (W.J.); hsl0213@126.com (S.H.), Tel.: +86-13607666083 (S.H.)

Table S1 List of differential metabolites involved in KEGG pathway in T12 vs CK stigmas

Compounds	Class	VIP	P-value	FC	Type	kegg_map
4-Hydroxybenzoic acid	Others	1.24	0.00	0.28	down	ko00130,ko00790,ko01100,ko01110,ko01240
Eugenol	Others	1.22	0.00	0.44	down	ko00940
Gluconolactone	Sugars	1.23	0.00	3.82	up	ko00030,ko01100,ko01110,ko01200
Indole-3-acetamide	Alkaloids	1.24	0.00	0.28	down	ko00380,ko01100
Pyridoxamine	Alkaloids	1.17	0.04	2.02	up	ko00750,ko01100
2-Acetamido-2-Deoxy-D-Mannopyranose	Sugars	1.23	0.01	0.03	down	ko00520,ko01100,ko01250
Cholestane-3,7,12,25-tetrol-3-glucuronide	Others	1.20	0.05	0.08	down	ko00040,ko00053,ko01100,ko01240
Guanine	Nucleotides	1.24	0.00	0.27	down	ko00230,ko01100,ko01232
Adenine	Nucleotides	1.24	0.00	0.26	down	ko00230,ko00908,ko01100,ko01110,ko01232

p-Coumaroylagmatine	Alkaloids	1.14	0.00	2.92	up	ko00999,ko01100,ko01110
Cytidine	Nucleotides	1.21	0.01	0.32	down	ko00240,ko01100,ko01232,ko02010
3-(Methylthio)propionic acid	Organic acids	1.23	0.00	3.52	up	ko00270,ko00920,ko01100
Nicotinic acid adenine dinucleotide	Nucleotides	1.22	0.00	0.49	down	ko00760,ko01100,ko01240
Imidazoleacetic acid	Alkaloids	1.13	0.00	5.29	up	ko00340,ko01100
L-Phenylalanine	Amino acids	1.22	0.01	3.69	up	ko00360,ko00400,ko00460,ko00470,ko00940, ko00960,ko00966,ko00970,ko00996,ko00999, ko01100,ko01110,ko01210,ko01230,ko02010
Indolocarbazole nitrogen derivative	Others	1.23	0.00	0.48	down	ko01100,ko01110
Chorismic acid	Organic acids	1.23	0.00	0.14	down	ko00130,ko00400,ko00790,ko00996,ko00999, ko01100,ko01110,ko01230,ko01240
Adenosine monophosphate	Nucleotides	1.23	0.00	0.31	down	ko00230,ko00908,ko01100,ko01110,ko01232, ko01240
Cyclic AMP	Nucleotides	1.20	0.00	0.49	down	ko00230,ko01100
Digalacturonate	Sugars	1.20	0.02	3.22	up	ko00040,ko01100,ko02010
Nicotinuric acid	Amino acids	1.19	0.00	4.36	up	ko00760
Adenosine-5'-diphosphate	Nucleotides	1.20	0.00	3.75	up	ko00190,ko00195,ko00230,ko00908,ko01100, ko01110,ko01232,ko01240
Adenosine	Nucleotides	1.23	0.00	0.33	down	ko00230,ko01100,ko01232,ko02010
Glutamine	Amino acids	1.23	0.00	0.47	down	ko00220,ko00230,ko00240,ko00250,ko00470, ko00630,ko00750,ko00910,ko00970,ko01100, ko01230,ko01232,ko01240,ko02010
7-Methylxanthine	Nucleotides	1.22	0.00	3.04	up	ko00232,ko01100,ko01110
Xanthosine-5'-monophosphate	Nucleotides	1.22	0.01	2.68	up	ko00230,ko01100,ko01110,ko01232
8-Demethyl-8-(dimethylamino)riboflavin	Others	1.23	0.00	0.43	down	ko01100,ko01110

16-Hydroxyhexadecanoic acid	Lipids	1.06	0.04	2.46	up	ko00073,ko01100
Phenylacetylglycine	Amino acids	1.24	0.00	11.49	up	ko00360
Thymidine	Nucleotides	1.22	0.02	5.64	up	ko00240,ko01100,ko01232
(-)-Sedamine	Others	1.22	0.00	0.31	down	ko00960
Taxifolin	Flavonoids	1.04	0.03	0.32	down	ko00941,ko01100,ko01110
Curcumin	Others	1.23	0.00	0.22	down	ko00945,ko01110
2-amino-4-oxovaleric acid	Organic acids	1.21	0.01	3.50	up	ko00470,ko01100
Diguanosine tetraphosphate	Nucleotides	1.19	0.00	17.95	up	ko00230,ko01100
Nebramine	Others	1.23	0.02	0.08	down	ko00524,ko01110
Agmatine	Alkaloids	1.21	0.00	3.42	up	ko00330,ko00999,ko01100,ko01110
Harmaline	Alkaloids	1.17	0.02	2.70	up	ko00901
Sphingosine 1-phosphate	Lipids	1.21	0.00	0.29	down	ko00600,ko01100
Carnitine C16:0	Alkaloids	1.20	0.03	0.16	down	ko00071,ko01212
4-Hydroxy-3-methoxycinnamaldehyde	Others	1.05	0.02	0.44	down	ko00940,ko01100,ko01110
D-(+)-Neopterin	Alkaloids	1.15	0.00	5.66	up	ko00790
Secologanin	Others	1.21	0.00	0.28	down	ko00901,ko00902,ko00950,ko01100,ko01110
LysoPC 16:0	Lipids	1.20	0.00	2.75	up	ko00564
1-(4Z,7Z,10Z,13Z,16Z,19Z-docosahexaenoyl)-sn-glycero-3-phosphocholine	Lipids	1.18	0.04	0.26	down	ko00564
L-alpha-Aminobutyric acid	Amino acids	1.22	0.01	2.47	up	ko00270,ko01100
L-Proline	Amino acids	1.23	0.00	2.10	up	ko00330,ko00332,ko00470,ko00970,ko01100,ko01110,ko01230,ko02010
3,4-Dihydroxybenzaldehyde	Others	1.12	0.02	2.51	up	ko00950
(S)-7-(((2-O-6-Deoxy-alpha-L-mannopyranosyl)-beta-D-	Flavonoids	1.12	0.02	0.38	down	ko00941

glucopyranosyl)oxy)-2,3-dihydro-5-hydroxy-2-(3-hydroxy-4-methoxyphenyl)-4H-1-benzopyran-4-one						
Inosinic acid	Nucleotides	1.19	0.02	3.03	up	ko00230,ko01100,ko01110,ko01232,ko01240
Phenylacetaldehyde	Others	1.07	0.03	0.50	down	ko00360,ko01100
Indole	Alkaloids	1.23	0.00	0.45	down	ko00380,ko00400,ko00402,ko00999,ko01100,ko01110
Guanosine	Nucleotides	1.24	0.00	0.28	down	ko00230,ko01100,ko01232,ko02010
Diethanolamine	Alkaloids	1.23	0.00	2.19	up	ko00564
Isonocardicin A	Organic acids	1.22	0.00	2.80	up	ko00261,ko01100,ko01110
LysoPC(20:5(5Z,8Z,11Z,14Z,17Z))	Lipids	1.17	0.00	0.17	down	ko00564
1-Palmitoyl-2-oleoyl-sn-glycero-3-phosphate	Others	1.03	0.02	0.31	down	ko00561,ko00564,ko01100,ko01110,ko04070
3-Methoxyanthranilate	Others	1.23	0.01	0.45	down	ko00380
4-(Phosphonooxy)-L-threonine	Amino acids	1.13	0.01	0.38	down	ko00750,ko01100,ko01240
dTDP-3-amino-2,3,6-trideoxy-D-threo-hexopyranos-4-ulose	Nucleotides	1.16	0.05	0.26	down	ko00523,ko01100,ko01110,ko01250
(2S)-2-(3-carboxypropanamido)-6-oxoheptanedioic acid	Organic acids	1.22	0.00	0.18	down	ko00300,ko01100,ko01230
cyclo-Dopa-glucuronylglucoside	Benzenoids	1.20	0.00	2.91	up	ko00965,ko01110
Gossypol	Others	1.24	0.00	4.10	up	ko00909,ko01110
3'-Aenylic Acid	Nucleotides	1.14	0.01	0.45	down	ko00230,ko01100
Dihydromacarpine	Alkaloids	1.13	0.01	0.21	down	ko00950,ko01100,ko01110
Geranyl diphosphate	Organic acids	1.18	0.01	0.36	down	ko00130,ko01100,ko01110,ko01240
Xylobiose	Sugars	1.20	0.02	2.59	up	ko02010
Pyridoxine	Others	1.24	0.00	0.32	down	ko00750,ko01100,ko01240

Uridine-5'-diphosphate	Nucleotides	1.19	0.00	0.25	down	ko00240,ko00908,ko01100,ko01232,ko01240
Uridine-5'-monophosphate	Nucleotides	1.22	0.03	0.24	down	ko00240,ko01100,ko01232,ko01240
Uridine	Nucleotides	1.19	0.04	0.33	down	ko00240,ko01100,ko01232,ko02010
L-Aspartic acid	Amino acids	1.22	0.01	0.32	down	ko00220,ko00250,ko00260,ko00261,ko00270, ko00300,ko00340,ko00410,ko00460,ko00470, ko00710,ko00760,ko00770,ko00970,ko00999, ko01100,ko01110,ko01200,ko01210,ko01230, ko01240,ko02010
D-Mannose	Sugars	1.24	0.00	4.52	up	ko00051,ko00052,ko00520,ko01100,ko01250, ko02010
2-Trimethylaminoethylphosphonic acid	Others	1.23	0.00	0.14	down	ko00440,ko01100
Desaminotyrosine	Others	1.17	0.04	0.27	down	ko01100
(2S)-2-Isopropylmalate	Organic acids	1.24	0.00	0.43	down	ko00290,ko00620,ko01100,ko01110,ko01210, ko01230
Naringenin	Flavonoids	1.24	0.00	5.00	up	ko00941,ko00943,ko01100,ko01110
Galactinol	Sugars	1.15	0.00	11.68	up	ko00052,ko01100
UDP-xylose	Nucleotides	1.24	0.00	0.20	down	ko00520,ko00908,ko01100,ko01250
Dihydrosanguinarine	Alkaloids	1.23	0.01	0.19	down	ko00950,ko01100,ko01110
alpha-D-Galactosamine 1-phosphate	Sugars	1.22	0.00	3.31	up	ko00520,ko01100,ko01250
Isopentenyl pyrophosphate	Organic acids	1.23	0.01	2.36	up	ko00900,ko01100,ko01110
2'-Deoxyguanosine-5'-diphosphate	Nucleotides	1.23	0.00	2.43	up	ko00230,ko01100,ko01232
1,4-Dihydroxy-2-naphthoic acid	Benzenoids	1.19	0.02	0.40	down	ko00130,ko01100,ko01110,ko01240
AICA ribonucleotide	Nucleotides	1.23	0.00	2.45	up	ko00230,ko00340,ko01100,ko01110
coproporphyrinogen III	Others	1.23	0.00	2.26	up	ko00860,ko01100,ko01110,ko01240
ADP-glucose	Nucleotides	1.21	0.00	0.01	down	ko00500,ko00520,ko01100,ko01110,ko01250

p-Coumaraldehyde	Others	1.24	0.00	3.51	up	ko00940,ko01100,ko01110
Rosmarinic acid	Others	1.22	0.00	2.04	up	ko00350,ko01100
Floionolic acid	Lipids	1.10	0.01	0.46	down	ko00073
Guanosine-5'-diphosphate	Nucleotides	1.21	0.00	2.90	up	ko00230,ko01100,ko01232,ko04144
D-inositol-4-phosphate	Others	1.21	0.03	0.25	down	ko00562,ko01100,ko04070
5,6,7,8-Tetrahydrosarcinapterin	Others	1.15	0.00	0.21	down	ko00790,ko01100,ko01200,ko01240
Uridine-5'-diphosphate-glucose	Nucleotides	1.23	0.00	0.40	down	ko00040,ko00052,ko00053,ko00500,ko00520,ko00524,ko00561,ko00908,ko01100,ko01110,ko01240,ko01250
Gentisate aldehyde	Others	1.24	0.00	0.21	down	ko00350,ko01100
Maleic acid	Organic acids	1.23	0.00	0.35	down	ko00350,ko00650,ko00760,ko01100
Salidroside	Others	1.24	0.00	2.93	up	ko00350,ko01100
1,2-Docosahexanoyl-sn-glycero-3-phosphocholine	Others	1.23	0.00	0.36	down	ko00564,ko00590,ko00591,ko00592,ko01100,ko01110
Mannose 6-phosphate	Sugars	1.22	0.01	0.37	down	ko00051,ko00520,ko01100,ko01110,ko01240,ko01250
(1S,4aS,7aS)-7-methyl-1-[(2S,3R,4S,5S,6R)-3,4,5-trihydroxy-6-(hydroxymethyl)tetrahydropyran-2-yl]oxy-1,4a,5,7a-tetrahydrocyclopenta[c]pyran-4-carboxylic acid	Others	1.21	0.00	0.35	down	ko00902,ko01110
5-Phosphonooxy-L-lysine	Amino acids	1.20	0.05	0.32	down	ko00310,ko01100
9,10-Dihydroxystearic acid	Lipids	1.22	0.01	0.42	down	ko00073
3-[(1R,2S,3S,5Z,7S,8S,9Z,13S,14Z,17R,18R)-3,13,17-tris(2-carboxyethyl)-2,7,18-tris(carboxymethyl)-1,2,5,7,12,12,15,17-	Organic acids	1.18	0.00	0.11	down	ko00860,ko01100,ko01240

octamethyl-3,8,13,18,19,22-hexahydrocorrin-8-yl]propanoic acid						
UDP-L-arabinofuranose	Nucleotides	1.13	0.01	0.36	down	ko00520,ko01100,ko01250
19-Hydroxytabersonine	Alkaloids	1.12	0.02	0.50	down	ko00901,ko01110
dTDP-3-acetamido-3,6-dideoxy-alpha-D-galactopyranose	Nucleotides	1.24	0.00	0.10	down	ko00523,ko01100,ko01110,ko01250
Trypanothione	Organic acids	1.19	0.00	0.48	down	ko00480,ko01100
Curcumin diglucoside	Others	1.19	0.00	7.70	up	ko00945,ko01110

Table S2 List of differential metabolites involved in T12 vs CK stigmas

**Compounds**	**Class**	**VIP**	**P-value**	**FC**	**Type**
Isodesmosine	Others	1.06	0.01	0.36	down
4-Hydroxybenzoic acid	Others	1.24	0.00	0.28	down
Eugenol	Others	1.22	0.00	0.44	down
Pterostilbene Phosphate	Benzenoids	1.11	0.02	4.10	up
Ser-Gln	Amino acids	1.20	0.00	0.50	down
2-Methoxynaphthalene	Benzenoids	1.16	0.01	2.85	up
Prolyl-Histidine	Amino acids	1.23	0.00	0.45	down
Gluconolactone	Sugars	1.23	0.00	3.82	up
2-Phenylphenol	Others	1.20	0.00	0.19	down
1-Naphthol	Others	1.23	0.01	0.29	down
Indole-3-acetamide	Alkaloids	1.24	0.00	0.28	down
7-Ethoxy-4-methylcoumarin	Benzenoids	1.22	0.00	0.35	down
Vinblastine	Alkaloids	1.17	0.01	0.50	down

Oleoyl Ethyl Amide	Others	1.23	0.00	0.32	down
Pyridoxamine	Alkaloids	1.17	0.04	2.02	up
Astragaloside III	Others	1.19	0.03	0.13	down
Ethyl N-acetyl-L-tyrosinate	Amino acids	1.21	0.01	0.07	down
Eutylone	Benzenoids	1.15	0.05	0.43	down
4,4'-Oxybis(benzenesulfonyl hydrazide)	Benzenoids	1.04	0.02	0.11	down
Nodinitib-1	Benzenoids	1.23	0.00	0.21	down
3-Deaza-2'-deoxyadenosine	Nucleotides	1.24	0.00	0.46	down
2-Methoxy-4-vinylphenol	Others	1.22	0.00	2.65	up
2-Acetamido-2-Deoxy-D-Mannopyranose	Sugars	1.23	0.01	0.03	down
Dihydrocarvone	Others	1.03	0.05	0.43	down
Histidylleucine	Amino acids	1.17	0.02	0.06	down
Riboprine	Nucleotides	1.23	0.00	0.45	down
Cholestane-3,7,12,25-tetrol-3-glucuronide	Others	1.20	0.05	0.08	down
1,2-Benzenedicarboxylic acid, 4,4'-oxybis-	Benzenoids	1.24	0.00	3.80	up
Glutethimide	Others	1.12	0.01	6.60	up
Methyl 3-(((2-((diaminomethylidene)amino)thiazol-4-yl)methyl)sulfanyl)propanoate	Organic acids	1.16	0.02	0.32	down
beta-CYCLODEXTRIN	Sugars	1.08	0.03	0.40	down
Pittoside A	Others	1.23	0.01	0.20	down
Aloeresin A	Flavonoids	1.12	0.01	2.05	up
2-Sulfamoylacetylphenol	Others	1.16	0.00	0.23	down
LPC(O-16:0/2:0)	Lipids	1.21	0.00	0.25	down
N-Nitrosothiazolidine	Others	1.23	0.00	0.29	down
Trimethoprim	Others	1.17	0.00	0.35	down

4-(2-Hydroxyethyl)piperazin-1-ylethanesulphonic acid	Benzenoids	1.13	0.04	0.07	down
alpha-Guanidinoglutaric acid	Organic acids	1.20	0.03	8.65	up
Guanine	Nucleotides	1.24	0.00	0.27	down
Adenine	Nucleotides	1.24	0.00	0.26	down
p-Coumaroylagmatine	Alkaloids	1.14	0.00	2.92	up
Specioside	Others	1.18	0.04	3.82	up
Glycophymoline	Others	1.22	0.00	0.39	down
(2S)-2-[[(1S)-1-carboxy-3-methylbutyl]carbamoylamino]pentanedioic acid	Organic acids	1.13	0.00	9.89	up
Tyr-Pro-Phe	Amino acids	1.22	0.00	0.26	down
3-Phenoxypropionic acid	Benzenoids	1.21	0.02	4.27	up
6-Methylnicotinamide	Alkaloids	1.15	0.01	0.45	down
Ethyl 4-(benzoylcarbamothioylamino)benzoate	Benzenoids	1.18	0.04	18.60	up
3-Hydroxy-3',4'-dimethoxyflavone	Flavonoids	1.24	0.00	0.09	down
Glu-Thr	Amino acids	1.16	0.00	0.23	down
Ala-Gly	Amino acids	1.21	0.00	0.07	down
Ethenodeoxyadenosine	Others	1.21	0.00	0.06	down
N-Hydroxy-4-aminobiphenyl	Benzenoids	1.12	0.00	0.13	down
Ser-His	Amino acids	1.21	0.00	3.22	up
Spathulenol	Others	1.23	0.00	2.15	up
Acanthoside D	Others	1.19	0.02	0.34	down
Moupinamide	Alkaloids	1.08	0.00	20.72	up
Ser-Ser-Ser	Amino acids	1.20	0.00	0.34	down
Cycloheximide	Others	1.23	0.00	0.18	down
Cytidine	Nucleotides	1.21	0.01	0.32	down

Salicylamide	Alkaloids	1.20	0.00	0.35	down
Pentoxifylline	Alkaloids	1.01	0.04	0.26	down
3-(Methylthio)propionic acid	Organic acids	1.23	0.00	3.52	up
Nicotinic acid adenine dinucleotide	Nucleotides	1.22	0.00	0.49	down
Imidazoleacetic acid	Alkaloids	1.13	0.00	5.29	up
Artemisinin	Others	1.23	0.00	2.55	up
L-Phenylalanine	Amino acids	1.22	0.01	3.69	up
Indolocarbazole nitrogen derivative	Others	1.23	0.00	0.48	down
Cyclomorusin	Flavonoids	1.23	0.00	0.30	down
Morusin	Flavonoids	1.12	0.01	0.42	down
Glycerol trihexanoate	Others	1.23	0.00	0.38	down
Cynarasaponin F	Others	1.21	0.00	0.08	down
2-Aminoisobutyric acid	Organic acids	1.13	0.01	3.80	up
Aniline	Alkaloids	1.23	0.00	0.39	down
Methylgingerol	Others	1.22	0.00	0.38	down
Chorismic acid	Organic acids	1.23	0.00	0.14	down
Cystathionine	Amino acids	1.23	0.00	0.31	down
Tarennoside	Others	1.14	0.01	0.36	down
Adenosine monophosphate	Nucleotides	1.23	0.00	0.31	down
(-)-Oleoside 11-methyl ester	Others	1.23	0.00	0.16	down
Cyclic AMP	Nucleotides	1.20	0.00	0.49	down
Methionyl-leucyl-phenylalanine	Amino acids	1.21	0.01	0.05	down
Ile-Gln	Amino acids	1.20	0.01	0.29	down
Digalacturonate	Sugars	1.20	0.02	3.22	up
Asp-Lys	Amino acids	1.20	0.00	0.39	down

O,O-Diethyl hydrogen thiophosphate	Others	1.24	0.00	8.00	up
Casimiroedine	Others	1.18	0.02	0.14	down
3,3-Dimethyl-1,2-dithiolane	Others	1.24	0.00	0.28	down
Nicotinuric acid	Amino acids	1.19	0.00	4.36	up
Adenosine-5'-diphosphate	Nucleotides	1.20	0.00	3.75	up
Adenosine	Nucleotides	1.23	0.00	0.33	down
Quipazine maleate	Benzenoids	1.23	0.00	0.15	down
S-nitrosoglutathione	Amino acids	1.12	0.00	0.25	down
Probenecid	Benzenoids	1.05	0.01	2.25	up
(E)-1,7-bis(4-hydroxyphenyl)hept-4-en-3-one	Others	1.23	0.01	0.36	down
Ergocornine	Alkaloids	1.22	0.00	2.26	up
4',5,7-Trimethoxyflavone	Flavonoids	1.19	0.00	0.23	down
Glutamine	Amino acids	1.23	0.00	0.47	down
Phomenone	Others	1.24	0.00	0.18	down
2,4-Bis(3-methylbut-2-en-1-yl)benzene-1,3,5-triol	Others	1.24	0.00	0.39	down
6H-Indolo(2,3-a)quinolizin-5-ium, 3-ethyl-7,12-dihydro-	Others	1.23	0.00	0.11	down
7-Methylxanthine	Nucleotides	1.22	0.00	3.04	up
D-xylulose	Sugars	1.20	0.01	4.69	up
5-(Tetradecyloxy)-2-furoic acid	Organic acids	1.23	0.00	0.35	down
diethyl 2,6-dimethyl-4-oxo-4h-pyran-3,5-dicarboxylate	Others	1.23	0.00	0.18	down
12S-HHT	Lipids	1.16	0.01	7.13	up
1-Cinnamoylpiperidine	Alkaloids	1.24	0.00	0.14	down
Nb-Feruloyltryptamine	Alkaloids	1.22	0.00	2.60	up
2-Phenylaminoadenosine	Nucleotides	1.18	0.01	3.45	up
Euscaphic acid	Others	1.10	0.02	2.10	up

Robustic acid	Organic acids	1.20	0.03	0.39	down
Gallacetophenone	Others	1.23	0.00	0.22	down
Anisatin	Others	1.23	0.00	0.49	down
Xanthosine-5'-monophosphate	Nucleotides	1.22	0.01	2.68	up
Hesperidin	Flavonoids	1.22	0.00	0.17	down
Benzoylecgonine	Benzenoids	1.22	0.00	0.40	down
Axillarin	Flavonoids	1.22	0.02	3.37	up
Irisxanthone	Flavonoids	1.08	0.02	0.30	down
8-Demethyl-8-(dimethylamino)riboflavin	Others	1.23	0.00	0.43	down
16-Hydroxyhexadecanoic acid	Lipids	1.06	0.04	2.46	up
1,11-Undecanedicarboxylic acid	Lipids	1.23	0.00	0.38	down
Phenylacetylglycine	Amino acids	1.24	0.00	11.49	up
Thymidine	Nucleotides	1.22	0.02	5.64	up
Aspirin	Organic acids	1.19	0.00	16.20	up
Mercaptopurine	Nucleotides	1.23	0.00	3.21	up
4-Hydroxy-3-methoxybenzenemethanol	Others	1.24	0.00	0.21	down
(-)-Sedamine	Others	1.22	0.00	0.31	down
Galbeta1,3GlcNAc	Sugars	1.05	0.03	0.48	down
Cytisine	Alkaloids	1.23	0.01	0.20	down
Lecanoric acid	Organic acids	1.10	0.00	0.12	down
Taxifolin	Flavonoids	1.04	0.03	0.32	down
4'-Methoxy-alpha-pyrrolidinopropiophenone	Others	1.19	0.04	0.32	down
Biosone	Others	1.22	0.00	49.73	up
Isoalantolactone	Others	1.23	0.00	6.44	up
Neopine	Benzenoids	1.20	0.01	0.16	down

Curcumin	Others	1.23	0.00	0.22	down
Gitoxin	Others	1.12	0.02	0.06	down
Corosolic acid	Others	1.20	0.00	2.29	up
Ruixianglangdusu B	Others	1.23	0.01	0.22	down
2-amino-4-oxovaleric acid	Organic acids	1.21	0.01	3.50	up
Anethole	Others	1.22	0.01	0.41	down
Diguanosine tetraphosphate	Nucleotides	1.19	0.00	17.95	up
N-Acetylpyrrolidine	Alkaloids	1.24	0.00	0.17	down
Methyl vanillate	Others	1.03	0.03	0.44	down
Asclepin	Others	1.18	0.04	7.41	up
Phe-Glu	Amino acids	1.19	0.00	0.21	down
Ochrolifuanine A	Alkaloids	1.20	0.00	14.71	up
3',5'-Cyclic IMP	Nucleotides	1.10	0.04	2.88	up
1-(2,6-Dihydroxy-4-methoxyphenyl)-3-(4-methoxyphenyl)propan-1-one	Others	1.12	0.01	2.05	up
N-Deacetylcolchicine	Alkaloids	1.16	0.00	3.88	up
Leu-Tyr-Leu	Amino acids	1.23	0.00	0.25	down
Chelerythrine	Alkaloids	1.23	0.00	0.40	down
Nebramine	Others	1.23	0.02	0.08	down
1-Propenyl propyl sulfide	Others	1.12	0.00	0.26	down
Prolyl-leucyl-glycine	Amino acids	1.24	0.00	0.40	down
Quinquenoside F1	Others	1.08	0.00	0.18	down
Glycyl-L-phenylalanine	Amino acids	1.03	0.00	11.44	up
N-Acetylcadaverine	Alkaloids	1.20	0.00	0.46	down
Amataine	Alkaloids	1.14	0.02	0.26	down

Agmatine	Alkaloids	1.21	0.00	3.42	up
Zidovudine	Nucleotides	1.24	0.00	0.23	down
2-(2-Naphthoxy)propionic acid methyl ester	Benzenoids	1.24	0.00	4.77	up
Harmaline	Alkaloids	1.17	0.02	2.70	up
2-Ethylidene-1,5-dimethyl-3,3-diphenylpyrrolidine	Benzenoids	1.23	0.00	0.40	down
Rotundine	Alkaloids	1.23	0.00	0.25	down
Nordihydrocapsaicin	Alkaloids	1.22	0.00	2.28	up
Salsolinol	Others	1.22	0.00	0.10	down
Scandoside methyl ester	Others	1.19	0.00	0.09	down
Sphingosine 1-phosphate	Lipids	1.21	0.00	0.29	down
2-[[(2S)-5-amino-2-azaniumyl-5-oxopentanoyl]amino]acetate	Amino acids	1.22	0.01	0.33	down
(9S)-9-Amino-9-deoxoerythromycin	Others	1.23	0.01	18.91	up
Scillipheosidin 3-[glucosyl-(1->2)-rhamnoside]	Others	1.17	0.01	0.48	down
n-(alpha-Linolenoyl) tyrosine	Amino acids	1.23	0.00	0.09	down
Phe-Phe	Amino acids	1.13	0.01	0.31	down
Coriamyrtin	Others	1.17	0.01	0.44	down
Carnitine C18:0((-)-Stearoylcarnitine)	Others	1.24	0.00	3.51	up
S-Lactoylglutathione	Organic acids	1.23	0.00	2.98	up
NCGC00384598-01_C30H42O8_(1bS,4aR,7bS,8R,9R,9aS)-4a,7b-Dihydroxy-3-(hydroxymethyl)-1,1,6,8-tetramethyl-9a-[(2-methylbutanoyl)oxy]-5-oxo-1a,1b,4,4a,5,7a,7b,8,9,9a-decahydro-1H-cyclopropa[3,4]benzo[1,2-e]azulen-9-yl (2E)-2-methyl-2-butenoate	Others	1.06	0.00	4.26	up
Catharanthine	Alkaloids	1.23	0.00	0.04	down

Cnicine	Others	1.08	0.00	0.25	down
Carnitine C16:0	Alkaloids	1.20	0.03	0.16	down
(8)-Gingerol	Others	1.20	0.00	0.29	down
4-Phenyl-2-butanol	Benzenoids	1.19	0.01	0.08	down
Leurosine	Alkaloids	1.23	0.01	0.32	down
Isopropyl linoleate	Others	1.24	0.00	0.18	down
(R)-(+)-alpha,alpha-Diphenyl-2-pyrrolidinemethanol	Benzenoids	1.22	0.00	0.39	down
Yayoisaponin C	Others	1.20	0.04	0.13	down
Tyr-Gly-Gly	Amino acids	1.20	0.00	2.46	up
(1R,6S)-bicyclo[4.1.0]heptane-7-carboxylic acid	Organic acids	1.21	0.00	0.49	down
Picrotin	Others	1.11	0.00	0.11	down
Methyl 2-amino-6-tert-butyl-4,5,6,7-tetrahydro-1-benzothiophene-3-carboxylate	Benzenoids	1.17	0.00	5.24	up
Methyllycaconitine Perchlorate, Delphinium sp.	Alkaloids	1.04	0.02	0.50	down
Fulvoplumierin	Others	1.02	0.01	0.33	down
Cinegalline	Alkaloids	1.24	0.00	130.87	up
4-Hydroxy-3-methoxycinnamaldehyde	Others	1.05	0.02	0.44	down
Solanine	Alkaloids	1.21	0.01	0.18	down
Glutamyl-phenylalanine	Amino acids	1.21	0.00	0.15	down
Homodihydrocapsaicin	Alkaloids	1.16	0.00	3.62	up
N-tetradecanoyl-L-Homoserine Lactone	Amino acids	1.23	0.00	0.16	down
2-Amino-3H-quinazolin-4-one	Others	1.15	0.02	4.96	up
Phenylalanylphenylalanine	Amino acids	1.22	0.02	0.38	down
D-(+)-Neopterin	Alkaloids	1.15	0.00	5.66	up
Piperlongumine	Alkaloids	1.10	0.00	8.06	up

Dihydromethysticin	Benzenoids	1.23	0.00	2.27	up
Methyl-tyrosine	Amino acids	1.10	0.01	0.31	down
1,3-Dicaffeoylquinic acid	Others	1.17	0.01	0.38	down
7,8-Dehydroastaxanthianthin	Others	1.22	0.03	0.11	down
Vobtusine	Alkaloids	1.21	0.00	0.43	down
2-Amino-6-ureidohexanoic acid	Amino acids	1.09	0.02	0.31	down
10-Hydroxycamptothecin	Alkaloids	1.08	0.02	2.87	up
Secologanin	Others	1.21	0.00	0.28	down
1,2-Dioctanoyl-sn-glycero-3-phosphocholine	Others	1.19	0.00	0.24	down
Ser-Leu-Ile-Gly-Lys-Val-amide	Amino acids	1.23	0.00	0.29	down
Ac-Asp-Glu-Val-Asp-pNA	Benzenoids	1.21	0.00	0.29	down
Pinostrobin chalcone	Flavonoids	1.22	0.01	2.89	up
trans-2-Octenoic acid	Organic acids	1.21	0.00	0.30	down
Acetochlor esa	Benzenoids	1.23	0.01	0.13	down
Gly-Gly-Gln	Amino acids	1.23	0.00	0.05	down
N6-Methyladenosine	Nucleotides	1.20	0.01	2.06	up
Psoralidin	Others	1.22	0.00	0.11	down
N-lactoyl-phenylalanine	Amino acids	1.18	0.00	0.12	down
1,2-Dimyristoyl-sn-glycero-3-phosphocholine	Others	1.21	0.00	0.27	down
Valdecoxib	Benzenoids	1.22	0.00	5.75	up
Erythrono-1,4-lactone	Others	1.20	0.00	6.37	up
(E,E)-4,4''-Bi(N-4-hydroxycinnamoylserotonin)	Others	1.19	0.00	6.35	up
LysoPC 16:0	Lipids	1.20	0.00	2.75	up
Dihydrodaidzein	Flavonoids	1.13	0.00	0.41	down
D-piperidine acid	Organic acids	1.23	0.00	0.37	down

Podorhizol beta-D-glucoside	Others	1.13	0.00	0.16	down
Koryoginsenoside R1	Others	1.17	0.01	0.42	down
Cassitoroside	Others	1.06	0.01	0.26	down
3,5-Dimethoxycinnamic acid	Organic acids	1.15	0.01	0.46	down
Oxymatrine	Alkaloids	1.17	0.02	0.22	down
Annohexocin	Organic acids	1.19	0.00	0.33	down
Digoxigenin	Others	1.21	0.01	0.33	down
2-Hydroxy-6-pentadecylbenzoic acid	Benzenoids	1.23	0.00	2.79	up
3-(Diphenylphosphino)-1-propylamine	Benzenoids	1.17	0.00	0.20	down
1-(4Z,7Z,10Z,13Z,16Z,19Z-docosahexaenoyl)-sn-glycero-3-phosphocholine	Lipids	1.18	0.04	0.26	down
Myriceric acid C	Others	1.20	0.01	0.12	down
3-[(2S,3R,4S,5S,6R)-4,5-dihydroxy-6-(hydroxymethyl)-3-[(2S,3R,4R,5R,6S)-3,4,5-trihydroxy-6-methyloxan-2-yl]oxyoxan-2-yl]oxy-2-(3,4-dihydroxyphenyl)-5-hydroxy-7-[(2S,3R,4R,5R,6S)-3,4,5-trihydroxy-6-methyloxan-2-yl]oxychromen-4-one	Flavonoids	1.23	0.01	0.28	down
beta-N-Methylamino-L-alanine	Amino acids	1.12	0.01	4.43	up
N-octanoylsphingosine 1-phosphate	Organic acids	1.23	0.01	0.14	down
cis-Zeatin riboside	Nucleotides	1.13	0.01	0.06	down
4-Nitroso-N-phenylaniline	Benzenoids	1.23	0.00	2.05	up
Terminalin	Organic acids	1.15	0.01	2.44	up
Metamfepramone	Benzenoids	1.23	0.01	0.32	down
Leurosidine	Alkaloids	1.17	0.02	0.45	down
Lyciumin D	Alkaloids	1.18	0.00	0.37	down

Galacturonic acid	Organic acids	1.18	0.04	0.16	down
L-alpha-Aminobutyric acid	Amino acids	1.22	0.01	2.47	up
Phenylpropanolamine	Alkaloids	1.23	0.01	0.30	down
3-Oxo-octanoic acid (2-oxo-tetrahydro-furan-3-YL)-amide	Amino acids	1.21	0.00	0.15	down
L-Proline	Amino acids	1.23	0.00	2.10	up
Solanesyl-PP	Others	1.22	0.00	0.26	down
5-Methyluridine	Nucleotides	1.22	0.00	2.55	up
2'-Hydroxy-5'-methylacetophenone	Benzenoids	1.13	0.01	0.28	down
Met-Met-Ala	Amino acids	1.23	0.00	0.18	down
Cannabisin-b	Others	1.19	0.04	0.39	down
Abscisic acid	Organic acids	1.16	0.00	0.49	down
S-Adenosylmethionine	Nucleotides	1.23	0.00	2.59	up
Cinnamic acid	Others	1.11	0.01	2.02	up
3,4-Dihydroxybenzaldehyde	Others	1.12	0.02	2.51	up
(4Ar,6aS,9aR)-1,8,8-trimethyl-2-oxo-1,4,4a,6a,7,9-hexahydropentaleno[1,6a-c]pyran-5-carboxylic acid	Others	1.17	0.01	4.78	up
2''-p-Coumarylastragalin	Flavonoids	1.23	0.01	0.40	down
Dianthramine	Benzenoids	1.20	0.05	0.16	down
5,7,8-Trimethoxy-3-(4-methoxyphenyl)chromen-4-one	Benzenoids	1.24	0.00	0.24	down
Dide-O-methylsimmondsin	Sugars	1.13	0.02	3.35	up
N8-Acetylspermidine	Amino acids	1.22	0.00	0.47	down
Midodrine	Benzenoids	1.18	0.01	0.36	down
Senecionine N-oxide	Alkaloids	1.22	0.00	0.50	down
5-(4-Nitrobenzoylamino)isophthalic acid	Benzenoids	1.15	0.00	6.65	up

(S)-7-(((2-O-6-Deoxy-alpha-L-mannopyranosyl)-beta-D-glucopyranosyl)oxy)-2,3-dihydro-5-hydroxy-2-(3-hydroxy-4-methoxyphenyl)-4H-1-benzopyran-4-one	Flavonoids	1.12	0.02	0.38	down
Alanine glutamate	Amino acids	1.23	0.00	0.12	down
Glycyl-l-glutamine	Amino acids	1.22	0.00	0.40	down
Inosinic acid	Nucleotides	1.19	0.02	3.03	up
Dioxybenzone	Benzenoids	1.23	0.01	0.25	down
Di-N-acetylchitobiose	Sugars	1.17	0.03	0.08	down
2'-Adenylic acid	Nucleotides	1.24	0.00	0.31	down
5-Methoxypodophyllotoxin	Others	1.16	0.00	0.13	down
N(4)-Acetylsulfadiazine	Benzenoids	1.22	0.00	2.06	up
Kanokoside B	Others	1.18	0.01	0.43	down
Sucrose acetate isobutyrate	Sugars	1.21	0.02	0.38	down
(-)-Epicatechin gallate	Flavonoids	1.22	0.00	0.41	down
2-([1,1'-Biphenyl]-4-yloxy)ethylamine	Benzenoids	1.20	0.00	0.20	down
N,N-dimethylsphingosine	Others	1.23	0.00	2.93	up
Phenylacetaldehyde	Others	1.07	0.03	0.50	down
2-Hydroxy-4-(methylthio)butyric acid	Organic acids	1.21	0.00	0.45	down
Indole	Alkaloids	1.23	0.00	0.45	down
3,4-Dimethoxyphenethylamine	Benzenoids	1.14	0.01	2.49	up
cyclic ADP-ribose	Sugars	1.22	0.00	2.76	up
(2R)-2,8-Dimethyl-2-[(4S,8S)-4,8,12-trimethyltridecyl]-3,4-dihydrochromen-6-ol	Others	1.22	0.00	2.38	up
Icaritin	Flavonoids	1.15	0.01	0.48	down
Hydroxymethyltolbutamide	Benzenoids	1.19	0.01	0.17	down

5-[(Pyridin-2-ylmethyl)thio]-1,3,4-thiadiazol-2-amine	Others	1.21	0.03	0.40	down
Acetaminosalol	Others	1.23	0.00	0.44	down
Guanosine	Nucleotides	1.24	0.00	0.28	down
16alpha,17alpha-Dihydroxyprogesterone acetophenide	Others	1.18	0.02	2.00	up
(6E)-Piperamide-C7:1	Alkaloids	1.15	0.00	0.06	down
5-(4-Iodophenyl)pentanoic acid	Organic acids	1.17	0.01	0.48	down
Luteolinidin	Flavonoids	1.21	0.01	0.38	down
Azaserine	Others	1.04	0.02	0.38	down
Methyl linoleate	Lipids	1.22	0.00	0.48	down
Methyl stearate	Lipids	1.05	0.01	0.25	down
Diethanolamine	Alkaloids	1.23	0.00	2.19	up
Cys-Asp-Pro-Gly-Tyr-Ile-Gly-Ser-Arg	Amino acids	1.04	0.00	0.13	down
Saikosaponin BK1	Others	1.03	0.02	0.34	down
5,7-Dimethoxyflavone	Flavonoids	1.21	0.00	0.22	down
(2E,4E)-2,4-Hexadienoic acid	Organic acids	1.14	0.01	0.45	down
9,9-Bis(4-amino-3-methylphenyl)fluorene	Benzenoids	1.15	0.00	3.24	up
Eplerenone	Others	1.19	0.02	0.10	down
1-(3,4-Dimethoxyphenyl)-2-(2-methoxyphenoxy)propane-1,3-diol	Others	1.24	0.00	0.23	down
4-Dihydroboldenone	Others	1.20	0.01	2.22	up
3-Phenylpropanal	Benzenoids	1.19	0.02	0.24	down
5-(Hydroxymethyl)-2(5H)-furanone	Others	1.06	0.04	2.03	up
5-(4-(2-(5-Ethylpyridin-2-yl)-2-hydroxyethoxy)benzyl)thiazolidine-2,4-dione	Others	1.04	0.04	3.05	up
Arg-Pro-Leu	Amino acids	1.23	0.00	0.43	down

Peonidin-3-O-beta-galactopyranoside	Flavonoids	1.23	0.01	0.21	down
Lys-Val-Gly-Asp-Thr	Amino acids	1.10	0.00	13.46	up
His-Gln-Ala	Amino acids	1.03	0.02	0.43	down
Met-Trp-His	Amino acids	1.20	0.00	3.76	up
Val-Tyr-Lys-Val-Leu	Amino acids	1.10	0.02	0.46	down
Arg-His-Thr	Amino acids	1.23	0.00	19.73	up
Leu-Ala-Arg-Glu-Leu	Amino acids	1.21	0.00	0.19	down
Lys-Val-Glu-Ile-Asp	Amino acids	1.22	0.01	0.35	down
Ile-His-Arg-Arg	Amino acids	1.12	0.01	0.30	down
Lys-Met-Glu	Amino acids	1.20	0.00	0.09	down
Asp-Val-Ala-Arg	Amino acids	1.24	0.00	0.20	down
Asp-Asp-Phe-Gln-Lys	Amino acids	1.19	0.00	0.33	down
Lys-Val-Ile-Arg	Amino acids	1.22	0.00	0.43	down
Arg-Phe-Thr	Amino acids	1.21	0.01	0.30	down
Gln-Phe-Glu-Lys	Amino acids	1.22	0.00	0.50	down
1,2-Dioctanoyl-sn-glycerol	Others	1.23	0.00	0.41	down
Arg-Cys-His	Amino acids	1.20	0.00	0.47	down
Met-Trp-Glu	Amino acids	1.22	0.00	0.38	down
Asp-Ser-Glu-Asp	Amino acids	1.13	0.01	0.39	down
Cys-Arg-Asp	Amino acids	1.19	0.00	0.44	down
Ile-Leu-Arg-Lys	Amino acids	1.22	0.00	0.26	down
Ile-Gln-Asn-Glu	Amino acids	1.11	0.01	0.45	down
Tyr-Phe-Asp-Arg	Amino acids	1.16	0.00	0.02	down
Thr-Glu-Phe-Asp	Amino acids	1.24	0.00	0.24	down
Asn-Ser-Glu-Asp-Thr	Amino acids	1.17	0.02	2.36	up

Thr-Ser-Gly	Amino acids	1.22	0.01	0.35	down
Trp-Met-Trp	Amino acids	1.18	0.00	2.38	up
Val-Asn-His-Val-Thr	Amino acids	1.20	0.01	0.40	down
Lys-Asn-Leu-Tyr-Thr	Amino acids	1.23	0.01	10.51	up
His-Asp-His	Amino acids	1.24	0.00	0.17	down
Leu-Tyr-Arg-Ile-Thr	Amino acids	1.11	0.01	0.41	down
His-His-Asn	Amino acids	1.24	0.00	0.18	down
Ile-Gln-Phe-Arg	Amino acids	1.23	0.01	0.29	down
Glu-Ser-Thr-Lys	Amino acids	1.20	0.01	0.26	down
Pro-Trp-Tyr	Amino acids	1.24	0.00	0.26	down
Tetrahydrogambogic acid	Organic acids	1.21	0.00	5.83	up
Phe-Lys-Leu-Arg	Amino acids	1.12	0.00	0.21	down
Gly-Phe-Gln	Amino acids	1.21	0.00	0.22	down
Ser-Asp-Thr-Glu	Amino acids	1.21	0.00	0.37	down
His-Glu-His	Amino acids	1.11	0.01	0.25	down
Gln-Glu-Ser-Gln-Asp	Amino acids	1.22	0.03	15.00	up
Asp-Ser-Ser	Amino acids	1.20	0.00	0.33	down
Met-Met-Asp	Amino acids	1.24	0.00	0.37	down
Asp-Glu-Phe	Amino acids	1.23	0.00	0.36	down
Tyr-Thr-Gln-Arg	Amino acids	1.21	0.02	0.31	down
His-Glu-Trp	Amino acids	1.24	0.00	0.18	down
Cys-Trp-Ala	Amino acids	1.21	0.00	0.18	down
Ile-Ala-Trp	Amino acids	1.21	0.00	0.50	down
His-Cys-Gln	Amino acids	1.24	0.00	0.36	down
Val-Leu-Asp-Phe-Glu	Amino acids	1.22	0.01	0.22	down

His-Phe-Arg	Amino acids	1.23	0.00	0.39	down
Asp-Glu-Arg	Amino acids	1.11	0.01	0.44	down
Tyr-Tyr-Leu	Amino acids	1.23	0.00	0.12	down
Gln-Gly-Tyr	Amino acids	1.23	0.00	0.24	down
His-Arg-Leu-Leu-Leu	Amino acids	1.15	0.01	0.41	down
Asp-Tyr-Met	Amino acids	1.23	0.00	0.34	down
Met-Phe-Trp	Amino acids	1.18	0.02	0.46	down
His-Pro-Trp	Amino acids	1.24	0.00	0.22	down
Ile-Tyr-Met	Amino acids	1.23	0.00	0.35	down
trans-Resveratrol 4'-O-glucuronide	Others	1.22	0.00	0.44	down
Inflatine	Alkaloids	1.13	0.02	2.62	up
Tyrosyl-Methionine	Amino acids	1.24	0.00	0.32	down
Phe-Phe-Arg	Amino acids	1.01	0.01	2.51	up
Ser-Ala-Arg	Amino acids	1.20	0.05	3.42	up
Glu-Val-Phe-Asp-Glu	Amino acids	1.23	0.01	0.16	down
Thr-Leu-Asp-Glu	Amino acids	1.16	0.00	16.79	up
Trp-His-Ile	Amino acids	1.22	0.00	0.12	down
Leu-Pro-Glu-Phe-Tyr	Amino acids	1.22	0.00	0.09	down
Phe-HoPhe-OH	Amino acids	1.23	0.00	0.30	down
Lys-Tyr-Val-Lys	Amino acids	1.19	0.00	0.48	down
Nonic acid	Organic acids	1.21	0.01	0.39	down
Gln-His-His	Amino acids	1.21	0.05	0.10	down
Pro-Glu-Glu-Leu-Lys	Amino acids	1.07	0.00	0.21	down
Asn-Leu	Amino acids	1.14	0.03	2.54	up
Trp-Phe	Amino acids	1.23	0.00	0.34	down

Asp-His-Leu-Thr-Gln	Amino acids	1.19	0.03	0.23	down
Lys-Asp-Thr-Lys	Amino acids	1.23	0.00	0.27	down
Pro-His-Phe-Asp-Lys	Amino acids	1.23	0.00	3.39	up
Thr-His-Tyr	Amino acids	1.23	0.00	0.21	down
Tyr-Glu-Lys-Thr-Tyr	Amino acids	1.24	0.00	0.06	down
Tyr-Val-Lys-Ala-Leu	Amino acids	1.15	0.00	0.28	down
Glu-Phe-Tyr-Tyr-Arg	Amino acids	1.23	0.01	0.10	down
Tyr-Met-Arg	Amino acids	1.24	0.00	0.10	down
Cyclocreatine	Nucleotides	1.23	0.00	0.16	down
Tyr-Leu-Gly-Lys-Arg	Amino acids	1.18	0.00	0.18	down
Val-Tyr-Leu-Arg	Amino acids	1.16	0.03	0.49	down
Trp-Glu-Glu	Amino acids	1.23	0.00	0.47	down
Glu-Gln-Leu-Val-Arg	Amino acids	1.20	0.00	0.33	down
Ile-Ser-Glu	Amino acids	1.24	0.00	3.40	up
Asp-Val-Ala	Amino acids	1.20	0.00	0.35	down
Ala-Ala-Ala-Arg-Phe	Amino acids	1.24	0.00	0.45	down
Ile-Lys-Arg-Asp-Phe	Amino acids	1.14	0.01	0.47	down
Phe-Glu-Ser-Phe-Gly	Amino acids	1.21	0.01	6.56	up
Asp-Cys-Trp	Amino acids	1.20	0.01	0.40	down
Arg-Trp-His	Amino acids	1.17	0.03	2.05	up
Leu-Leu-Leu-Ser-Leu	Amino acids	1.15	0.04	0.48	down
Asp-Trp-Arg	Amino acids	1.23	0.00	2.21	up
Val-Gln-Phe-Asp	Amino acids	1.13	0.00	0.37	down
Asp-Asp-His	Amino acids	1.23	0.00	5.67	up
2,3-Methylenedioxypyrovalerone	Benzenoids	1.13	0.00	9.75	up

Arg-Tyr-Val-Lys	Amino acids	1.23	0.01	0.41	down
Tyr-Ile	Amino acids	1.10	0.05	9.75	up
Asp-Thr-Gln-Arg	Amino acids	1.22	0.00	6.17	up
Thr-His-Gly	Amino acids	1.20	0.00	0.36	down
Glu-Phe-Tyr-Leu-Gly	Amino acids	1.21	0.00	0.04	down
Thr-Arg-Gln-Glu	Amino acids	1.17	0.03	0.33	down
Trp-Phe-Asp	Amino acids	1.21	0.00	0.27	down
Lys-Abu-OH	Amino acids	1.21	0.01	0.04	down
His-Tyr-Arg	Amino acids	1.07	0.00	0.16	down
Daphnoretin	Others	1.22	0.00	2.79	up
Pro-Lys-Lys-Val-Gly	Amino acids	1.20	0.02	0.42	down
Arg-Ala-Leu	Amino acids	1.22	0.00	43.08	up
Val-Thr-Tyr-Asp-Met	Amino acids	1.07	0.00	19.87	up
Met-Ser-Phe-Thr-Phe	Amino acids	1.16	0.00	0.11	down
Asp-Ile-Gln	Amino acids	1.21	0.01	0.39	down
Lys-Gly-Ala-Cys-Lys	Amino acids	1.11	0.01	0.15	down
Met-Glu-Trp	Amino acids	1.18	0.00	0.36	down
Pro-Tyr-Asp	Amino acids	1.22	0.01	14.34	up
Thr-Ile-Lys	Amino acids	1.18	0.00	4.86	up
Phe-Val-Asn	Amino acids	1.21	0.01	0.06	down
Gln-Thr-Met	Amino acids	1.22	0.00	2.89	up
Leu-Ala-Pro-Lys-Ile	Amino acids	1.17	0.02	0.30	down
Cys-Glu-His	Amino acids	1.00	0.03	0.36	down
Asp-Met-His	Amino acids	1.10	0.01	2.65	up
Asn-Gln-Phe	Amino acids	1.24	0.00	0.21	down

peonidin pentose	Flavonoids	1.20	0.03	0.46	down
Ser-Leu-Gln	Amino acids	1.13	0.03	0.46	down
Phe-Glu-Ser	Amino acids	1.13	0.00	0.13	down
His-Gly-Lys-Lys-Val	Amino acids	1.20	0.01	0.45	down
Trp-Tyr-Gln	Amino acids	1.23	0.00	0.10	down
Phe-Leu-Leu-Val-Phe	Amino acids	1.22	0.00	0.38	down
Trp-Asn-Cys	Amino acids	1.23	0.00	0.43	down
Pro-Val-Val	Amino acids	1.21	0.03	0.16	down
Lys-Thr-Ile-Thr-Leu	Amino acids	1.17	0.00	15.23	up
Met-Asp-Ser	Amino acids	1.24	0.00	0.37	down
Lys-Asp-Tyr-Glu-Leu	Amino acids	1.20	0.00	0.35	down
Neu5Ac2-3[GalNAc1-4]Gal1-4GlcSp	Sugars	1.11	0.04	0.33	down
His-Leu-OH	Amino acids	1.20	0.00	7.23	up
Gly-Val-Leu	Amino acids	1.20	0.00	0.44	down
Pro-Asp-Leu	Amino acids	1.22	0.01	2.75	up
Arg-Glu-Arg-Val-Tyr	Amino acids	1.09	0.01	0.41	down
Asn-Val-His-Asp	Amino acids	1.24	0.00	0.24	down
7,3'-Dihydroxy-8,2',4'-trimethoxyisoflavan	Others	1.14	0.00	0.33	down
Glu-Arg-His-Asp	Amino acids	1.09	0.00	0.04	down
His-Gly-Phe-His-Val	Amino acids	1.24	0.00	0.16	down
Trp-His-His	Amino acids	1.21	0.00	6.92	up
Met-Ala-Glu	Amino acids	1.18	0.02	0.31	down
Pro-Gly-Glu-Cys-Leu	Amino acids	1.24	0.00	3.43	up
Lys-Leu-Ala	Amino acids	1.20	0.01	2.20	up
Phe-Phe-Ser	Amino acids	1.20	0.00	0.11	down

Leu-Gly-Asp-Val-Ile	Amino acids	1.19	0.00	2.49	up
Val-Ala-Ser	Amino acids	1.22	0.00	0.41	down
Leu-Ala-Pro-Leu-Glu	Amino acids	1.23	0.01	0.19	down
Trp-Trp-Asp	Amino acids	1.21	0.01	0.22	down
Tyr-Glu-Asp-Tyr-Val	Amino acids	1.22	0.00	2.78	up
Cys-Ala-Pro-Pro-Thr	Amino acids	1.15	0.04	2.02	up
Lys-His-Ala-Val-Ser	Amino acids	1.20	0.03	0.40	down
Tyr-Pro-Thr-Val-Asn	Amino acids	1.23	0.02	0.15	down
Phe-Gln-Asp-Arg	Amino acids	1.22	0.00	0.19	down
Gln-Gln-Gln-Glu-Gln	Amino acids	1.22	0.00	2.85	up
Phe-Ala-His	Amino acids	1.17	0.00	0.40	down
His-Phe-Cys	Amino acids	1.24	0.00	0.38	down
Phe-Gln-Phe-Phe-Lys	Amino acids	1.10	0.00	0.19	down
Arachidonic acid-biotin	Others	1.21	0.00	0.18	down
Phe-Phe-Met	Amino acids	1.24	0.00	0.32	down
Met-Asp-Met	Amino acids	1.11	0.01	3.56	up
Thr-Glu-Met	Amino acids	1.14	0.00	0.13	down
Trp-Ser-His	Amino acids	1.23	0.00	0.38	down
Met-Glu-Leu-Ser-Glu	Amino acids	1.23	0.01	0.11	down
Leu-Ser-Ser-Asp-Ile	Amino acids	1.20	0.00	0.36	down
Ile-Tyr-Val-Arg	Amino acids	1.19	0.00	38.23	up
Leu-Thr-Thr-Glu-Glu	Amino acids	1.24	0.00	0.10	down
Met-Cys-Asp-Phe-Thr	Amino acids	1.19	0.02	0.30	down
Ser-Met-Met	Amino acids	1.21	0.00	0.40	down
Ser-His-Glu-Ala-Glu	Amino acids	1.16	0.02	0.30	down

Pro-Asp-Glu	Amino acids	1.20	0.03	0.16	down
Azacyclonol	Benzenoids	1.23	0.00	0.40	down
Gln-Gln-Trp	Amino acids	1.22	0.00	0.29	down
Gly-Arg-Ser-Asp-Asp	Amino acids	1.16	0.02	2.18	up
Asp-Met-Leu-Asp-Leu	Amino acids	1.06	0.01	0.44	down
Ala-Phe-Gln-Lys	Amino acids	1.16	0.00	3.48	up
Phe-Tyr-Gln	Amino acids	1.17	0.02	0.44	down
Tyr-Tyr-Gln	Amino acids	1.23	0.01	0.44	down
Glu-Phe-Glu	Amino acids	1.20	0.00	0.14	down
Ser-Ser-Asn	Amino acids	1.23	0.00	0.27	down
Leu-Arg-Asp-Lys	Amino acids	1.22	0.00	0.46	down
Leu-Gly-Arg	Amino acids	1.20	0.00	0.34	down
Glu-Gln-Lys-Asp-Arg	Amino acids	1.23	0.00	0.16	down
Glu-Glu-Met-Ile-Ala	Amino acids	1.23	0.00	0.28	down
Pro-Pro-Ser	Amino acids	1.22	0.00	5.14	up
Ala-Phe-Lys	Amino acids	1.19	0.00	0.38	down
His-Trp-Glu	Amino acids	1.24	0.00	0.45	down
Glu-Tyr-Gly	Amino acids	1.16	0.03	7.19	up
His-Ala-Arg	Amino acids	1.17	0.01	2.10	up
Ala-Asn-Asp-Asp	Amino acids	1.21	0.01	0.14	down
3,6,11,12-Tetrakis(acetyloxy)-5-(4-hydroxyphenyl)-8-oxatricyclo[7.4.0.0^{2,7}]trideca-1(13),2,4,6,9,11-hexaen-4-yl acetate	Organic acids	1.23	0.01	3.79	up
Aziridyl benzoquinone	Benzenoids	1.20	0.00	0.29	down
Pgp(18:3(6Z,9Z,12Z)/22:6(4Z,7Z,10Z,13Z,16Z,19Z))	Others	1.15	0.00	2.26	up

5-(3',4',5'-Trihydroxyphenyl)-gamma-valerolactone-3'-O-sulphate	Organic acids	1.21	0.00	2.85	up
[(2R,3S,4S,5R,6S)-3,4,5-trihydroxy-6-[(6Z)-1-hydroxy-6-(2-hydroxy-2-methylpropylidene)-2,2,4,4-tetramethyl-3,5-dioxocyclohexyl]oxyoxan-2-yl]methyl 3,4,5-trihydroxybenzoate	Benzenoids	1.23	0.00	0.22	down
3,4,5-trihydroxy-6-(5-hydroxy-1-oxo-1H-isochromene-3-carbonyloxy)oxane-2-carboxylic acid	Organic acids	1.23	0.01	2.87	up
N-Acetyl-S-(N-allylthiocarbamoyl)-L-cysteine	Amino acids	1.23	0.00	3.50	up
D-erythritol 1-phosphate	Organic acids	1.23	0.00	3.49	up
1D-myo-inositol 2-acetamido-2-deoxy-alpha-D-glucopyranoside 3-phosphate	Organic acids	1.17	0.00	0.30	down
Militarinone D	Others	1.19	0.03	0.17	down
1-Isobutanol	Others	1.16	0.01	0.39	down
PA(22:6(4Z,7Z,10Z,13Z,16Z,19Z)/22:6(4Z,7Z,10Z,13Z,16Z,19Z))	Others	1.23	0.03	0.09	down
Met-Met	Amino acids	1.11	0.02	0.44	down
Puberanine	Others	1.09	0.01	2.19	up
PE-NMe2(22:6(4Z,7Z,10Z,13Z,16Z,19Z)/22:6(4Z,7Z,10Z,13Z,16Z,19Z))	Others	1.23	0.00	0.28	down
castasterone-23-O-glucoside	Others	1.12	0.02	0.11	down
9,12,13-Trihydroxy-10,15-octadecadienoic acid	Lipids	1.18	0.00	0.49	down
trans-Acenaphthen-1,2-diol	Benzenoids	1.23	0.01	25.36	up
CL(8:0/8:0/8:0/10:0)	Others	1.22	0.00	0.47	down
GSK-3beta Peptide Inhibitor	Benzenoids	1.13	0.00	0.29	down

PGP(a-13:0/a-17:0)	Others	1.11	0.02	0.37	down
LPC(0:0/18:3)	Lipids	1.22	0.01	0.37	down
TRIETHYL PHOSPHATE	Organic acids	1.23	0.00	3.42	up
6-{[7-({6-[(acetyloxy)methyl]-3-{[3,4-dihydroxy-4-(hydroxymethyl)oxolan-2-yl]oxy}-4,5-dihydroxyoxan-2-yl}oxy)-2-(4-hydroxyphenyl)-4-oxo-4H-chromen-5-yl]oxy}-3,4,5-trihydroxyoxane-2-carboxylic acid	Organic acids	1.12	0.04	0.40	down
N-(2-((N-(2-((N-(2-((N-(2-Hydroxypropanoyl)valyl)oxy)-3-methylbutanoyl)valyl)oxy)propanoyl)valyl)oxy)-3-methylbutanoyl)valine	Amino acids	1.11	0.01	0.48	down
(R)-Byakangelicin 2'-glucoside	Others	1.17	0.04	2.25	up
CDP-dipalmitoyl-sn-glycerol	Others	1.06	0.02	0.31	down
Dioleoyl phosphatidate	Others	1.19	0.05	0.25	down
1,2-Octadienoylglycine	Organic acids	1.04	0.01	0.41	down
3,4,5-trihydroxy-6-[2-hydroxy-4-(3,5,6,7-tetrahydroxy-4-oxo-4H-chromen-2-yl)phenoxy]oxane-2-carboxylic acid	Organic acids	1.16	0.00	0.16	down
Thelehantin H	Benzenoids	1.06	0.00	5.96	up
Hoodigoside E	Others	1.23	0.00	0.42	down
Isonocardicin A	Organic acids	1.22	0.00	2.80	up
2-amino-4-oxo-5,6,7,8-tetrahydro-1H-pteridine-6-carboxylate	Others	1.18	0.01	0.35	down
N-[3-[(2-oxo-1-naphthalenylidene)methylamino]phenyl]-2-phenylpropanamide	Benzenoids	1.17	0.00	3.05	up
[(1R,2S,3E,7R,8E,10R,13S)-9,10,13-triacetyloxy-2,7-dihydroxy-8,12,15,15-tetramethyl-5-oxo-4-bicyclo[9.3.1]pentadeca-3,8,11-trienyl]methyl acetate	Others	1.14	0.03	3.84	up

Disinomenine	Alkaloids	1.24	0.01	0.10	down
Biotinate	Others	1.18	0.00	2.51	up
(4S,10aS)-3-(Ethoxymethyl)-4beta,6beta,10alpha-triacetoxy-6,9alpha-dimethyl-6aalpha,9-dihydroxy-4,5,6,6a,7,8,9,10-octahydro-2H-naphtho[8a,1-b]furan-2-one	Benzenoids	1.18	0.00	2.23	up
6-[[2-[4-[3-[3,4-dihydroxy-4-(1H-indole-3-carbonyloxymethyl)oxolan-2-yl]oxy-4,5-dihydroxy-6-(hydroxymethyl)oxan-2-yl]oxyphenyl]-4-oxo-2,3-dihydrochromen-7-yl]oxy]-3,4,5-trihydroxyoxane-2-carboxylic acid	Organic acids	1.08	0.01	2.90	up
N-[1-(4-methoxy-6-oxopyran-2-yl)-2-methylbutyl]acetamide	Others	1.01	0.02	0.22	down
N-acetyl-L-citrullinate	Amino acids	1.21	0.00	4.35	up
omega-Sulfo-beta-dihydromenaquinone-9	Others	1.03	0.02	0.45	down
1-Naphthaldehyde	Benzenoids	1.21	0.00	0.24	down
CID 14605151	Benzenoids	1.22	0.00	4.18	up
Asn-Met-Met	Amino acids	1.19	0.03	0.18	down
D-Fructose	Sugars	1.13	0.02	0.49	down
LysoPC(20:5(5Z,8Z,11Z,14Z,17Z))	Lipids	1.17	0.00	0.17	down
[4-(5,7-dihydroxy-6,8-dimethyl-4-oxo-3,4-dihydro-2H-1-benzopyran-2-yl)phenyl]oxidanesulfonic acid	Organic acids	1.15	0.00	0.19	down
2-Acetyl-3-lauryl-3',4-di(isovaleryl)sucrose	Others	1.11	0.00	0.25	down
CID 131769968	Benzenoids	1.09	0.00	5.16	up
15-Epibetanidin 5-[E-feruloyl-(->3)-apiosyl-(1->2)-glucoside]	Alkaloids	1.05	0.00	0.14	down
Ser-Asn-Val-Ser-Asp	Amino acids	1.22	0.03	0.11	down
Pro-Trp-Gln	Amino acids	1.22	0.00	0.27	down

PS(22:5(4Z,7Z,10Z,13Z,16Z)/22:6(4Z,7Z,10Z,13Z,16Z,19Z))	Others	1.23	0.00	0.28	down
Met-Tyr-Val	Amino acids	1.22	0.01	2.07	up
N-acetyl-D-glucosaminyldiphosphodolichol(2-)	Organic acids	1.02	0.00	0.18	down
6-Methoxy-2-octaprenyl-1,4-benzoquinone	Benzenoids	1.14	0.01	0.41	down
CID 131835430	Others	1.21	0.02	0.22	down
[(4-hydroxyphenyl)(2,4,6-trihydroxy-3-oxo-2,3-dihydro-1-benzofuran-2-yl)methoxy]sulfonic acid	Organic acids	1.20	0.00	4.06	up
PS(20:1(11Z)/22:6(4Z,7Z,10Z,13Z,16Z,19Z))	Others	1.16	0.00	0.19	down
1-(Naphthalen-2-yl)-2-(pyrrolidin-1-yl)pentan-1-one	Benzenoids	1.15	0.01	2.38	up
Leu-Nap-OH	Amino acids	1.23	0.01	0.27	down
Val-Ser-Gln	Amino acids	1.24	0.00	2.46	up
6-({2,4-dihydroxy-2-[hydroxy(4-hydroxyphenyl)methyl]-3-oxo-2,3-dihydro-1-benzofuran-6-yl}oxy)-3,4,5-trihydroxyoxane-2-carboxylic acid	Organic acids	1.14	0.01	0.08	down
1,3,4-Thiadiazole-2-ethylamine	Others	1.24	0.00	2.79	up
6-[(3-Aminophenyl)methyl]-4,6-dihydro-4-methyl-2-(methylsulfinyl)-5h-thieno[2',3':4,5]pyrrolo[2,3-d]pyridazin-5-one	Others	1.21	0.00	4.78	up
NCGC00384573-01_C24H28O11_2-Propenoic acid, 3-(4-hydroxyphenyl)-, [(1S,4aR,5S,7aS)-1-(beta-D-glucopyranosyloxy)-1,4a,5,7a-tetrahydro-5-hydroxycyclopenta[c]pyran-7-yl]methyl ester, (2E)-	Benzenoids	1.16	0.01	5.70	up
1-Palmitoyl-2-oleoyl-sn-glycero-3-phosphate	Others	1.03	0.02	0.31	down
Phe-Lys-Tyr	Amino acids	1.22	0.00	0.02	down
1-Heptadecanoyl-glycero-3-phosphate	Others	1.14	0.01	2.05	up
3-Methoxyanthranilate	Others	1.23	0.01	0.45	down

(E)-4-[[(2S)-3-amino-2-azaniumyl-3-oxopropyl]amino]-4-oxobut-2-enoate	Others	1.21	0.00	0.17	down
3-(2-Methylbutanoyl)-4-(isovaleryl)sucrose	Others	1.24	0.00	3.37	up
Ser-Asp-Asn	Amino acids	1.22	0.00	4.29	up
Ile-Ile-Trp	Amino acids	1.18	0.02	0.18	down
PE-NMe2(20:1(11Z)/15:0)	Others	1.12	0.01	0.25	down
His-Lys-Leu-Val-Val	Amino acids	1.22	0.00	0.34	down
2,3-Bis(4-hydroxyphenyl)propionitrile	Benzenoids	1.18	0.01	0.44	down
(1R,4R,5R,7R,8R,11R,17S,18S)-4,5,7,8,17-pentahydroxy-14,18-dimethyl-6-methylidene-3,10-dioxapentacyclo[9.8.0.01,7.04,19.013,18]nonadec-14-ene-9,16-dione	Others	1.07	0.02	0.32	down
Pro-Gln-Gln-Asp-Glu	Amino acids	1.21	0.01	0.47	down
Lys-His-Ile-Glu-Glu	Amino acids	1.13	0.00	12.74	up
Glu-Val-Phe-Glu	Amino acids	1.23	0.00	0.26	down
(2S)-2,3-dihydroxy-1-(4-hydroxy-3,5-dimethoxyphenyl)propan-1-one	Others	1.20	0.00	0.19	down
Trp-Glu-Cys	Amino acids	1.24	0.00	0.09	down
Asp-Ile-Met	Amino acids	1.19	0.01	0.29	down
Asp-Ile-Lys-Glu-Met	Amino acids	1.24	0.00	0.18	down
Leu-Met-His	Amino acids	1.20	0.00	0.43	down
Val-His-Phe-Glu	Amino acids	1.22	0.00	2.79	up
Carnitine C10:2	Others	1.14	0.00	0.36	down
6-Hydroxy-fad	Nucleotides	1.10	0.02	0.30	down
5-methyltetrahydropteroyltri-L-glutamate	Amino acids	1.15	0.04	22.36	up

Petunidin 3-(6''-p-coumarylglucoside)	Flavonoids	1.08	0.01	0.07	down
Val-Pro-Glu-Pro-Lys	Amino acids	1.23	0.00	0.15	down
2-Benzyl-heptanoic acid	Organic acids	1.22	0.02	8.18	up
p-Cresol glucuronide	Benzenoids	1.23	0.01	2.68	up
Pro-Ile-His-Asp-Arg	Amino acids	1.18	0.00	0.04	down
Asn-Asp-Tyr-Asp	Amino acids	1.23	0.00	0.42	down
Ile-Glu-Tyr-Cys-Lys	Amino acids	1.20	0.00	0.14	down
1-O-Hexadecyl-2-arachidonoyl-sn-glycero-3-phosphocholine	Benzenoids	1.00	0.05	0.43	down
Glu-Lys-His-Cys-Arg	Amino acids	1.10	0.01	2.77	up
2-(3,4-dihydroxyphenyl)-5-hydroxy-3-[(3,4,5-trihydroxy-6-methyloxan-2-yl)oxy]-7-[(3,4,5-trihydroxy-6-{[(3,4,5-trihydroxy-6-methyloxan-2-yl)oxy]methyl}oxan-2-yl)oxy]-4H-chromen-4-one	Others	1.23	0.00	0.45	down
Arg-Gln-Asp-Glu-His	Amino acids	1.19	0.00	0.17	down
Trp-Glu-Met	Amino acids	1.11	0.04	0.12	down
PI(18:0/20:4(5Z,8Z,11Z,14Z))	Others	1.12	0.01	0.35	down
Phe-Ser-Leu-Phe-Asp	Amino acids	1.19	0.00	3.64	up
(-)-Threo-isodihomocitric acid	Organic acids	1.11	0.01	0.42	down
Glu-Val-Tyr-Asp	Amino acids	1.22	0.00	0.29	down
2-(formamido)-N1-(5-phospho-beta-D-ribosyl)acetamidine	Organic acids	1.23	0.00	6.42	up
Thr-Tyr-Glu-Lys-Tyr	Amino acids	1.20	0.00	0.49	down
[(2R)-2-pentadecanoyloxy-3-phosphonooxypropyl] (Z)-octadec-11-enoate	Others	1.23	0.00	0.21	down
Leu-Arg-Gly-Pro-Ser	Amino acids	1.19	0.02	0.44	down

(+)-Catechin-3-O-beta-D-gluco(2,6-biscinnamoyl)-pyranoside	Others	1.23	0.00	0.24	down
Gal1-4[Fuc1-3]GlcNAcSp	Sugars	1.22	0.00	0.39	down
Tyr-Leu-Pro-Leu-Arg-Phe-NH2	Amino acids	1.22	0.01	0.32	down
Tyr-Cys	Amino acids	1.22	0.00	4.17	up
2-[(2R,4aS,8S,8aS)-8-[2-[(4aS,7R,8aR)-7-(1-carboxyethenyl)-1-hydroxy-4a-methyl-2-oxo-6,7,8,8a-tetrahydro-5H-naphthalen-1-yl]ethyl]-4a-methyl-7-oxo-1,2,3,4,8,8a-hexahydronaphthalen-2-yl]prop-2-enoic acid	Organic acids	1.20	0.00	6.30	up
CID 85372017	Benzenoids	1.23	0.01	3.53	up
Rhodomyrtone, >=95% (LC/MS-ELSD)	Benzenoids	1.20	0.02	0.27	down
Chromopyrrolate	Organic acids	1.03	0.03	3.69	up
Carnitine C8:1(2-Octenoylcarnitine)	Others	1.21	0.01	4.09	up
Ser-Tyr-Trp	Amino acids	1.24	0.00	0.24	down
Gly-Tyr-Met	Amino acids	1.23	0.00	0.25	down
1-Myristoyl-2-(1-enyl-stearoyl)-sn-glycero-3-phosphoethanolamine	Others	1.24	0.00	0.24	down
Glu-Ile-Asp-Thr-Lys	Amino acids	1.11	0.01	12.60	up
Thr-Cys-Gly	Amino acids	1.08	0.02	0.34	down
[(3S,4R,5R)-3,4-dihydroxy-5-[[(2R,3S,4S,5R,6R)-3,4,5-trihydroxy-6-[2-(4-hydroxyphenyl)ethoxy]oxan-2-yl]methoxy]oxolan-3-yl]methyl 3,4,5-trimethoxybenzoate	Benzenoids	1.23	0.02	20.17	up
PC(O-16:0/O-1:0)	Lipids	1.18	0.02	0.47	down
Malyngamide J	Others	1.01	0.04	0.39	down
Gly-Tyr-Leu	Amino acids	1.23	0.00	0.48	down

Ile-Arg-Ile-Ile-Val	Amino acids	1.03	0.01	0.10	down
3-Hydroxy-4-methylanthranilate	Benzenoids	1.13	0.01	0.47	down
Curtisian E	Benzenoids	1.03	0.03	0.32	down
Val-Leu-Arg	Amino acids	1.24	0.00	0.29	down
(3S,7R)-11-methoxy-3,7-dimethyl-6,8,16,20-tetraoxapentacyclo[10.8.0.02,9.03,7.013,18]icosa-1,4,9,11,13(18)-pentaene-17,19-dione	Others	1.22	0.00	0.24	down
Gly-Leu-Met	Amino acids	1.17	0.04	0.44	down
Phe-Val-Asp	Amino acids	1.23	0.00	111.62	up
Neocarrabiose	Others	1.18	0.03	7.28	up
GalNAca1-3[Fuca1-2]Galb1-4[Fuca1-3]GlcNAcb-Sp	Sugars	1.17	0.00	6.57	up
Asp-Trp-Asp	Amino acids	1.18	0.00	0.15	down
Pusilatin C	Others	1.22	0.00	4.71	up
({1-[2-(2,4-dihydroxyphenyl)-7-hydroxy-8-(3-methylbut-2-en-1-yl)-4-oxo-4H-chromen-3-yl]-3,7-dimethylocta-2,6-dien-4-yl}oxy)sulfonic acid	Others	1.24	0.00	0.21	down
Tyr-Asp-Ala-Asp	Amino acids	1.22	0.01	0.08	down
Asp-Gly-His-Ser-Thr	Amino acids	1.12	0.01	2.14	up
Vitexin-2''-O-glucoside	Flavonoids	1.22	0.01	0.35	down
LysoPA(i-19:0/0:0)	Others	1.23	0.01	6.48	up
Phe-Tyr-Phe-Lys-Ile	Amino acids	1.19	0.05	0.24	down
Ile-Val-Ile-Phe-Asn	Amino acids	1.02	0.01	2.94	up
Ala-His-Ile	Amino acids	1.21	0.01	0.43	down
Arg-Phe-Lys	Amino acids	1.17	0.00	0.28	down
Lys-Gly-Phe	Amino acids	1.20	0.04	0.14	down

Ser-Ile-Gln	Amino acids	1.24	0.00	0.40	down
Glu-Pro-Gly-Tyr-Ser	Amino acids	1.00	0.02	0.28	down
N,N-Diethyl-2-cyano-3-(3,4-dihydroxy-5-nitrophenyl)propanamide	Others	1.18	0.00	3.55	up
His-Val-Arg-Asp	Amino acids	1.22	0.00	0.04	down
Phe-Met-Gly	Amino acids	1.23	0.00	0.33	down
Met-Tyr-Cys	Amino acids	1.24	0.00	0.50	down
Met-Val-Gln	Amino acids	1.23	0.01	0.19	down
[[(E)-3-methyl-5-[(1R,2S)-1,2,5,5-tetramethyl-2,3,6,7,8,8a-hexahydronaphthalen-1-yl]pent-2-enoxy]-oxidophosphoryl] phosphate	Organic acids	1.14	0.01	2.16	up
Asp-Glu-Tyr	Amino acids	1.04	0.04	2.03	up
6-[2-(3-{3-[(3,3-dimethyloxiran-2-yl)methyl]-2,4,6-trihydroxyphenyl}-3-oxopropyl)-5-hydroxyphenoxy]-3,4,5-trihydroxyoxane-2-carboxylic acid	Organic acids	1.23	0.00	7.57	up
Phe-Trp	Amino acids	1.17	0.00	2.44	up
2,2-Diallylpiperazine	Others	1.18	0.00	0.32	down
Glu-Val-Ser	Amino acids	1.19	0.04	3.21	up
Ala-Pro-Val	Amino acids	1.23	0.00	0.28	down
Arg-Leu-Lys	Amino acids	1.17	0.02	0.38	down
Pro-Asn-Ala-Leu-Ser	Amino acids	1.20	0.00	3.09	up
Ser-Glu-Ile-Glu	Amino acids	1.12	0.01	0.31	down
6-[(6,7-dihydroxy-4-oxo-3-phenyl-3,4-dihydro-2H-1-benzopyran-5-yl)oxy]-3,4,5-trihydroxyoxane-2-carboxylic acid	Organic acids	1.22	0.00	0.41	down

(2R,3S,4S,5R,6R)-2-(hydroxymethyl)-6-[4-[(E)-3-hydroxyprop-1-enyl]-2-methoxyphenoxy]oxane-3,4,5-triol	Others	1.22	0.01	6.54	up
CID 45783223	Others	1.21	0.00	0.39	down
Leu-Ser-Asp	Amino acids	1.24	0.00	4.73	up
Bendazac	Organic acids	1.15	0.01	0.44	down
[(1S,2S,3aR,5S,6E,10R,11S,13R,13aR)-3a,10,11,13-tetraacetyloxy-2,5,8,8-tetramethyl-12-methylidene-4,9-dioxo-1,2,3,5,10,11,13,13a-octahydrocyclopenta[12]annulen-1-yl] acetate	Others	1.23	0.00	0.34	down
3-{[3,4-dihydroxy-5-(hydroxymethyl)oxolan-2-yl]oxy}-5,7-dihydroxy-2-(3-hydroxyphenyl)-4H-chromen-4-one	Others	1.24	0.00	0.34	down
Hematoxylin	Others	1.23	0.00	0.12	down
Leu-Phe-Ser	Amino acids	1.24	0.00	0.36	down
Deoxygedunol acetate	Organic acids	1.16	0.05	0.42	down
6-{[4,7-dihydroxy-3-(2-hydroxy-4-methoxyphenyl)-3,4-dihydro-2H-1-benzopyran-5-yl]oxy}-3,4,5-trihydroxyoxane-2-carboxylic acid	Organic acids	1.23	0.00	0.08	down
Asp-Glu-Val-Phe-Asp	Amino acids	1.17	0.01	3.44	up
Asp-Ala-Phe	Amino acids	1.23	0.00	0.45	down
Annoglaxin	Others	1.18	0.00	0.08	down
Trp-Thr	Amino acids	1.24	0.00	0.21	down
Met-Lys-Asn-His-Leu	Amino acids	1.23	0.00	0.21	down
Ile-His-Val-Leu-Glu	Amino acids	1.22	0.00	0.47	down
3,4,5-trihydroxy-6-{5-hydroxy-2-[5-hydroxy-7-methoxy-6-(3-methylbut-1-en-1-yl)-3-(3-methylbut-2-en-1-yl)-4-oxo-4H-chromen-2-yl]phenoxy}oxane-2-carboxylic acid	Organic acids	1.11	0.02	2.45	up

[5-(3,5-dihydroxy-7-methoxy-4-oxo-4H-chromen-2-yl)-2-hydroxyphenyl]oxidanesulfonic acid	Organic acids	1.24	0.00	2.64	up
NCGC00384655-01_C31H38O11_7,11-Methano-5H-cyclodeca[3,4]benz[1,2-b]oxet-5-one, 6,12b-bis(acetyloxy)-12-(benzoyloxy)-1,2a,3,4,4a,6,9,10,11,12,12a,12b-dodecahydro-4,9,11-trihydroxy-4a,8,13,13-tetramethyl-, (2aR,4aS,6R,9S,11S,12S,12aR,12bS)-	Benzenoids	1.24	0.00	0.32	down
Glu-Tyr-Gln	Amino acids	1.11	0.01	0.19	down
Indolmycenic acid	Alkaloids	1.17	0.00	2.73	up
Asn-Trp-Glu	Amino acids	1.13	0.01	0.41	down
Phe-Ala-Thr-Asp-Asp	Amino acids	1.14	0.02	0.47	down
Carnitine C14:2	Others	1.17	0.00	0.04	down
Azelnidipine	Benzenoids	1.22	0.00	0.45	down
4-(Phosphonooxy)-L-threonine	Amino acids	1.13	0.01	0.38	down
6-[1-(3,4-dihydroxyphenyl)-3-(4-methoxy-1-benzofuran-5-yl)-1,3-dioxopropan-2-yl]-3,4,5-trihydroxyoxane-2-carboxylic acid	Organic acids	1.08	0.01	0.19	down
dTDP-3-amino-2,3,6-trideoxy-D-threo-hexopyranos-4-ulose	Nucleotides	1.16	0.05	0.26	down
N-Succinyl-2-amino-6-oxopimelate	Organic acids	1.23	0.00	0.12	down
1,2-Dipalmitoleoyl-sn-glycero-3-phosphoethanolamine	Others	1.23	0.01	0.25	down
Trp-Met-Tyr	Amino acids	1.15	0.00	0.19	down
6-{4-[3-(3,7-dimethylocta-2,6-dien-1-yl)-7-hydroxy-8-(4-hydroxy-3-methylbut-2-en-1-yl)-4-oxo-4H-chromen-2-yl]-3-hydroxyphenoxy}-3,4,5-trihydroxyoxane-2-carboxylic acid	Organic acids	1.23	0.01	0.38	down

3,4,5-Trihydroxy-6-[[3,4,5-trihydroxy-6-[[2-(2-hydroxypropan-2-yl)-7-oxo-2,3-dihydrouro[3,2-g]chromen-9-yl]oxy]oxan-2-yl]methoxy]oxane-2-carboxylic acid	Organic acids	1.19	0.00	0.10	down
4-(3,4'-dihydroxy-4,4,7,8a-tetramethyl-6'-oxospiro[2,3,4a,5,6,7-hexahydro-1H-naphthalene-8,2'-3,8-di	Organic acids	1.17	0.04	11.96	up
Ser-Phe-Asp-Lys-Ser	Amino acids	1.23	0.00	0.13	down
3,4,5-trihydroxy-6-(3-hydroxy-5-methoxybenzoyloxy)oxane-2-carboxylic acid	Organic acids	1.21	0.00	0.23	down
NCGC00381046-01_C41H66O14_9,19-Cyclolanost-24-en-26-oic acid, 3-[(2-O-hexopyranosylpentopyranosyl)oxy]-12,15-dihydroxy-, (3beta,8xi,9beta,24E)-	Organic acids	1.22	0.01	0.11	down
Ala-Ser-Ser	Amino acids	1.18	0.03	0.28	down
(2S)-2-(3-carboxypropanamido)-6-oxoheptanedioic acid	Organic acids	1.22	0.00	0.18	down
1-Alkyl-2-acylglycerophosphoethanolamine	Others	1.21	0.00	3.85	up
Gln-Leu-Lys	Amino acids	1.02	0.01	0.18	down
6,8-bis[3,7-dihydroxy-2-(3-hydroxyphenyl)-3,4-dihydro-2H-1-benzopyran-4-yl]-2-(3-hydroxyphenyl)-3,4-dihydro-2H-1-benzopyran-3,5,7-triol	Benzenoids	1.05	0.01	0.29	down
N-(Cyclopropylmethyl)-7-[3,5-dihydroxy-2-(3-hydroxy-5-phenylpent-1-EN-1-YL)cyclopentyl]hept-5-enamide	Organic acids	1.19	0.00	6.36	up
Ochrindole D	Others	1.08	0.01	2.26	up
Met-Tyr	Amino acids	1.22	0.00	4.68	up
8,16-Dihydroxy-6-(6-hydroxy-1-benzouran-2-yl)-12-methyl-3,13-dioxapentacyclo[10.7.1.02,10.04,9.014,19]icosa-2(10),4,6,8,14(19),15,17-heptaen-11-one	Lipids	1.20	0.02	2.89	up
TG(12:0/10:0/10:0)	Others	1.20	0.02	0.24	down

Ala-Asn-Val-Asp	Amino acids	1.22	0.03	0.16	down
Phe-Phe-Trp	Amino acids	1.23	0.00	0.47	down
Carnitine C20:1 Isomer 1	Others	1.21	0.00	0.46	down
2-amino-4-[(2-{[2-carboxy-2-hydroxy-1-(2-hydroxyphenyl)ethyl]sulfanyl}-1-[(carboxymethyl)-C-hydroxycarbonimidoyl]ethyl)-C-hydroxycarbonimidoyl]butanoic acid	Organic acids	1.23	0.00	0.39	down
Tyr-Phe	Amino acids	1.23	0.00	28.08	up
Thr-Leu-Pro-Pro-Gly	Amino acids	1.15	0.00	0.25	down
Asn-Phe-Val	Amino acids	1.21	0.04	0.07	down
Di-O-Glucosylquinic acid	Others	1.21	0.03	4.01	up
Gly-Tyr-Asn-Ser-Tyr	Amino acids	1.23	0.00	0.19	down
Ser-Pro-Phe	Amino acids	1.19	0.00	0.33	down
Cys-Phe-Ala	Amino acids	1.23	0.00	0.40	down
2-(2-hydroxy-3,4-dimethoxyphenyl)-1-(2,4,6-trihydroxyphenyl)propan-1-one	Others	1.21	0.00	4.82	up
Nororientalinium(1+)	Others	1.19	0.00	6.03	up
Val-Thr-Leu-Asp-Met	Amino acids	1.22	0.00	0.27	down
Nap-Trp-OH	Amino acids	1.06	0.01	0.41	down
Val-Asp-Tyr-Val-Leu	Amino acids	1.21	0.00	0.29	down
Trp-Gln-Gln-Asp-Glu	Amino acids	1.15	0.01	0.31	down
6-{[2-(2,4-dihydroxyphenyl)-3-(3-hydroxy-3-methylbutyl)-8,8-dimethyl-4-oxo-4H,8H-pyrano[2,3-f]chromen-5-yl]oxy}-3,4,5-trihydroxyoxane-2-carboxylic acid	Organic acids	1.23	0.01	0.23	down

3,4,5-trihydroxy-6-(3-hydroxy-4-{7-hydroxy-3-[(2Z)-4-hydroxy-3-(4-methylpent-3-en-1-yl)but-2-en-1-yl]-8-(3-methylbut-2-en-1-yl)-4-oxo-4H-chromen-2-yl}phenoxy)oxane-2-carboxylic acid	Organic acids	1.22	0.03	0.10	down
Tyr-Leu-Glu	Amino acids	1.20	0.00	0.07	down
Gly-Gln-Trp	Amino acids	1.24	0.00	0.37	down
Luteolin 7-O-(2-apiosyl-4-glucosyl-6-malonyl)-glucoside	Flavonoids	1.03	0.01	3.67	up
Asn-Leu-Pro-Ala-Lys	Amino acids	1.20	0.03	0.39	down
3,4,5-trihydroxy-6-{4-[1-hydroxy-3-(5-methoxy-2,2-dimethyl-2H-chromen-6-yl)propyl]phenoxy}oxane-2-carboxylic acid	Organic acids	1.14	0.04	0.28	down
Arg-Glu-Val	Amino acids	1.12	0.02	0.44	down
Tyr-Glu-Val-Lys	Amino acids	1.23	0.00	0.15	down
Ala-Trp-Ser	Amino acids	1.16	0.00	0.30	down
Asp-Asn	Amino acids	1.09	0.00	7.00	up
Phe-Val-Ile	Amino acids	1.18	0.04	0.31	down
Thr-Asn-Val	Amino acids	1.23	0.01	4.73	up
Gal1-3GlcNAc1-3Gal1-4GlcNAcSp	Sugars	1.23	0.01	0.23	down
Cannabinol	Others	1.22	0.01	0.18	down
Asn-Ser-Thr	Amino acids	1.14	0.02	2.39	up
Trp-Tyr-Met	Amino acids	1.22	0.01	0.29	down
Asn-Gly-Thr	Amino acids	1.20	0.00	0.46	down
Aspartylasparagine	Amino acids	1.01	0.02	5.09	up
Gly-Phe-Asn	Amino acids	1.16	0.00	24.10	up
(S,Z)-N-(2-Oxotetrahydrofuran-3-yl)tetradec-9-enamide	Others	1.23	0.01	2.97	up

Carnitine C9:2-OH	Others	1.24	0.00	0.15	down
Val-Tyr-Asp	Amino acids	1.24	0.00	9.43	up
Pyridinium-3,5-dicarboxylate mononucleotide	Nucleotides	1.22	0.01	8.44	up
Ser-Ile-Ala-Asp	Amino acids	1.21	0.01	0.43	down
His-Tyr-Gln	Amino acids	1.23	0.00	2.87	up
Asp-Tyr-Ile-Glu	Amino acids	1.05	0.04	0.14	down
Myxochromide S3	Others	1.05	0.04	3.31	up
LPE(0:0/18:3)	Others	1.07	0.03	0.41	down
(6aR,9R,10aR)-N-[(1S,2S,4R,7S)-2-hydroxy-7-methyl-5,8-dioxo-4-propan-2-yl-3-oxa-6,9-diazatricyclo[7.3.0.02,6]dodecan-4-yl]-7-methyl-6,6a,8,9,10,10a-hexahydro-4H-indolo[4,3-fg]quinoline-9-carboxamide	Alkaloids	1.21	0.00	3.12	up
3-(2,4-Dihydroxyphenyl)-5-hydroxy-6-(2-hydroxy-3-methylbut-3-enyl)-8,8-dimethylpyrano[2,3-h]chromen-4-one	Others	1.14	0.04	0.15	down
Leu-Gly-Ala-Pro-Gly	Amino acids	1.23	0.00	2.47	up
Gly-Glu-Asp	Amino acids	1.14	0.00	0.30	down
2,3-Dihydroxypropyl 2-[(octadec-9-enoyl)amino]ethyl hydrogen phosphate	Lipids	1.18	0.01	0.41	down
Ethisterone	Others	1.23	0.01	2.37	up
Asp-Glu-Ile-Asp	Amino acids	1.21	0.00	0.49	down
(2E,6E,10E,14E)-geranylfarnesyl diphosphate	Organic acids	1.20	0.00	0.26	down
17-(4-hydroxyphenyl)heptadecanoyl-AMP	Nucleotides	1.13	0.03	0.18	down
Ala-Phe-Asn	Amino acids	1.19	0.00	2.03	up
Megalomicin C1	Others	1.22	0.00	0.28	down

1-pentadecanoyl-2-(13Z,16Z-docosadienoyl)-glycero-3-phosphate	Others	1.16	0.02	0.11	down
Ala-Tyr-Trp	Amino acids	1.22	0.00	0.22	down
S-(p-Azidophenacyl)glutathione	Amino acids	1.15	0.00	3.70	up
5-Ammoniopentanal	Others	1.18	0.03	15.63	up
3,4-dihydroxy-5-{[(2E)-3-[4-hydroxy-3-(sulfooxy)phenyl]prop-2-enoyl]oxy}cyclohex-1-ene-1-carboxylic acid	Organic acids	1.07	0.01	0.46	down
cyclo-Dopa-glucuronylglucoside	Benzenoids	1.20	0.00	2.91	up
1,2-di-(9Z-hexadecenoyl)-sn-glycero-3-phospho-(1'-sn-glycerol)	Others	1.12	0.00	0.18	down
Phe-Ile-Val-Gly-Asp	Amino acids	1.19	0.01	49.93	up
Tyr-Gln-Cys	Amino acids	1.22	0.02	0.27	down
2-O-Galloylgalactaric acid	Others	1.11	0.00	0.24	down
Gln-Asp-Cys-Tyr-Ser	Amino acids	1.23	0.00	0.01	down
TG(16:0/16:0/18:3(9Z,12Z,15Z))	Others	1.20	0.02	0.17	down
3,5-Dihydroxybenzoic acid	Organic acids	1.21	0.02	2.39	up
Kalbreclasine	Alkaloids	1.23	0.00	0.13	down
Epigallocatechin gallate	Flavonoids	1.23	0.01	0.34	down
(10E,12Z)-9-HODE	Lipids	1.23	0.00	0.44	down
Lysyl threonine	Amino acids	1.01	0.00	0.07	down
Sarothralin	Benzenoids	1.24	0.00	3.26	up
1,3-Dimethyluric acid	Amino acids	1.02	0.02	0.33	down
Isochamaejasmin	Flavonoids	1.04	0.01	3.07	up
1,2'-Di-O-galloylhamamelofuranose	Benzenoids	1.22	0.00	2.58	up

Gossypol	Others	1.24	0.00	4.10	up
Biflorin	Others	1.08	0.01	0.41	down
Erythorbic acid	Others	1.21	0.03	0.25	down
Heterophylliin A	Others	1.22	0.01	2.21	up
(6S)-2-methyl-6-[(1S)-4-methylidenecyclohex-2-en-1-yl]hept-2-en-4-one	Others	1.23	0.01	2.07	up
3'-Aenylic Acid	Nucleotides	1.14	0.01	0.45	down
11H-Benzofuro(3,2-b)(1)benzopyran-11-one, 2-(5,6-dihydro-9-hydroxy-2-methyl-2,6-methano-2H-1-benzoxocin-4-yl)-5a,10a-dihydro-1,3,5a,8-tetrahydroxy-10a-(3-methyl-2-butenyl)-	Others	1.17	0.00	0.26	down
Prim-O-glucosylcimifugin	Others	1.23	0.00	4.70	up
Triphenylphosphine oxide	Benzenoids	1.23	0.00	2.01	up
Taxifolin 3-O-acetate	Flavonoids	1.18	0.01	0.38	down
Okadaic acid	Organic acids	1.18	0.00	0.18	down
Dihydromacarpine	Alkaloids	1.13	0.01	0.21	down
Chalcomoracin	Others	1.13	0.01	2.02	up
Geranyl diphosphate	Organic acids	1.18	0.01	0.36	down
13-Hydroxy-6,9,11-octadecatrienoic acid	Lipids	1.24	0.00	0.31	down
Byssochlamic acid	Benzenoids	1.20	0.00	2.01	up
Triptophenolide	Others	1.20	0.00	3.14	up
Gloriosine	Benzenoids	1.21	0.01	0.37	down
Simplexoside	Others	1.22	0.00	2.38	up
Granatin A	Others	1.21	0.00	29.91	up
Manniflavanone	Others	1.17	0.00	6.18	up

Spinosin	Flavonoids	1.23	0.00	3.83	up
Trp-Asp	Amino acids	1.23	0.00	0.23	down
Agecorynin C	Benzenoids	1.23	0.00	10.95	up
3-(2,4-Cyclopentadien-1-ylidene)pregn-4-en-20-one	Others	1.00	0.01	0.26	down
Pentitol	Sugars	1.18	0.00	0.21	down
Kolaflavanone	Others	1.19	0.01	0.41	down
Xylobiose	Sugars	1.20	0.02	2.59	up
Madlongiside C	Others	1.19	0.00	0.24	down
Proglumide	Others	1.23	0.00	2.20	up
N-Demethylconoduramine B640243K425	Alkaloids	1.19	0.00	0.19	down
Dl-Lyxose	Sugars	1.20	0.02	0.35	down
Pyridoxine	Others	1.24	0.00	0.32	down
aureusidin 6-O-beta-glucoside	Flavonoids	1.17	0.05	0.47	down
4',6-Dihydroxyflavone	Flavonoids	1.22	0.01	0.21	down
2,3,3-Triphenylacrylonitrile	Others	1.22	0.00	0.44	down
Falaconitine	Benzenoids	1.24	0.00	0.26	down
Paptga	Benzenoids	1.24	0.00	2.25	up
Edetic acid	Organic acids	1.24	0.00	0.22	down
Dihydroergotamine	Alkaloids	1.20	0.00	2.20	up
Neoschaftoside	Flavonoids	1.19	0.03	0.33	down
Ruspolinone	Others	1.22	0.01	0.43	down
Phosphine-biotin	Benzenoids	1.05	0.00	5.88	up
Tyrosyl-tryptophan	Amino acids	1.18	0.01	0.30	down
3,2'-Dihydroxyflavone	Flavonoids	1.20	0.02	2.69	up
alpha,beta-Trehalose	Sugars	1.20	0.00	0.45	down

5,7-Dihydroxy-2-(4-hydroxy-3-methoxy-phenyl)-3,6,8-trimethoxy-chromen-4-one	Flavonoids	1.22	0.00	0.28	down
Uridine-5'-diphosphate	Nucleotides	1.19	0.00	0.25	down
Uridine-5'-monophosphate	Nucleotides	1.22	0.03	0.24	down
Uridine	Nucleotides	1.19	0.04	0.33	down
Pomiferin	Flavonoids	1.22	0.00	0.14	down
5,7,3',4'-Tetrahydroxy-6,8-dimethoxyflavone	Flavonoids	1.23	0.01	2.71	up
Sulfinpyrazone	Benzenoids	1.19	0.00	0.49	down
D-(-)-Fructose	Sugars	1.21	0.01	0.45	down
L-Aspartic acid	Amino acids	1.22	0.01	0.32	down
Thr-His	Amino acids	1.21	0.01	2.35	up
Nifuradene	Others	1.23	0.01	0.27	down
Glycylmethionine	Amino acids	1.23	0.01	3.80	up
Isorhoifolin	Others	1.24	0.00	0.17	down
Nicotinate adenine dinucleotide phosphate	Nucleotides	1.23	0.00	2.80	up
Nebularine	Nucleotides	1.13	0.01	0.49	down
Digallic acid	Others	1.20	0.01	3.84	up
Cellobiotol	Others	1.22	0.00	0.26	down
D-Mannose	Sugars	1.24	0.00	4.52	up
1-Octadecyl Lysophosphatidic Acid	Others	1.23	0.00	10.32	up
D-(+)-Cellobiose	Sugars	1.23	0.00	22.47	up
3-(Methylthio)heptanal	Others	1.22	0.00	0.40	down
2-Trimethylaminoethylphosphonic acid	Others	1.23	0.00	0.14	down
2-(Diphenylphosphino)benzaldehyde	Benzenoids	1.23	0.00	0.12	down
6-(3,3-DMA)chrysin	Flavonoids	1.21	0.00	0.50	down

Desaminotyrosine	Others	1.17	0.04	0.27	down
Rhapontigenin	Others	1.22	0.02	4.99	up
Aspulvinone E	Others	1.23	0.00	4.33	up
(2S)-2-Isopropylmalate	Organic acids	1.24	0.00	0.43	down
Naringenin	Flavonoids	1.24	0.00	5.00	up
Obamegine	Others	1.21	0.00	2.77	up
Isoeugenitol	Others	1.12	0.00	4.01	up
Talampicillin	Others	1.22	0.00	0.48	down
Isochondrodendrine	Benzenoids	1.20	0.00	0.29	down
Luteic acid	Organic acids	1.20	0.00	3.45	up
Ellagic acid	Others	1.23	0.01	5.56	up
5'-Deoxyadenosine	Nucleotides	1.23	0.00	0.41	down
Brazilin	Flavonoids	1.15	0.01	0.34	down
Betulinic acid	Others	1.11	0.00	5.40	up
1-Kestose	Sugars	1.23	0.00	0.29	down
Clerodin	Others	1.23	0.01	22.17	up
Met-Lys	Amino acids	1.24	0.00	0.21	down
S-isopropyl isothiourea hydrobromide	Others	1.24	0.00	0.38	down
2-Methylhippuric acid	Amino acids	1.22	0.00	0.15	down
4'-Methoxyflavone	Flavonoids	1.18	0.01	0.06	down
Glycitin	Flavonoids	1.21	0.00	2.17	up
Inumakilactone A glycoside	Others	1.22	0.00	2.42	up
(1S,2S,4R)-p-Menth-8-ene-1,2,10-triol 2-glucoside	Others	1.21	0.00	2.65	up
(5a,6a,8a,11a)-8-Hydroxy-2-oxo-1(10),3-guaiadien-12,6-olide-15-al 8-(4-hydroxyphenylacetate)	Others	1.15	0.00	2.70	up

Galactinol	Sugars	1.15	0.00	11.68	up
UDP-xylose	Nucleotides	1.24	0.00	0.20	down
Dihydrosanguinarine	Alkaloids	1.23	0.01	0.19	down
alpha-D-Galactosamine 1-phosphate	Sugars	1.22	0.00	3.31	up
D-Nmappd	Benzenoids	1.23	0.00	0.25	down
Isopentenyl pyrophosphate	Organic acids	1.23	0.01	2.36	up
Hesperetin dihydrochalcone	Others	1.14	0.00	0.31	down
Piscidic acid	Others	1.24	0.00	3.75	up
2'-Deoxyguanosine-5'-diphosphate	Nucleotides	1.23	0.00	2.43	up
[(1R,2R,6S,7S,8R,10S,11S,12R,14S,16S,17R,18R)-6,7-dihydroxy-8-(hydroxymethyl)-4,18-dimethyl-5-oxo-14-phenyl-16-prop-1-en-2-yl-9,13,15,19-tetraoxahexacyclo[12.4.1.01,11.02,6.08,10.012,16]nonadec-3-en-17-yl] (2E,4E)-5-phenylpenta-2,4-dienoate	Others	1.02	0.04	0.31	down
[(1R,4Z,6R,7S)-4-ethenyl-17-hydroxy-6,7,14-trimethyl-3,8-dioxo-2,9-dioxa-14-azoniatricyclo[9.5.1.014,17]heptadeca-4,11-dien-7-yl] acetate	Alkaloids	1.24	0.00	8.00	up
Silicristin	Flavonoids	1.18	0.01	0.30	down
Isomalathion	Others	1.18	0.00	2.08	up
8-Desoxygartanin	Others	1.11	0.01	0.48	down
1,4-Dihydroxy-2-naphthoic acid	Benzenoids	1.19	0.02	0.40	down
AICA ribonucleotide	Nucleotides	1.23	0.00	2.45	up
Phenanthro[10,1-bc]pyran-5,11(1H,4H)-dione, 2,3,3a,6abeta,7,7aalpha,8,11a,11balpha,11c-decahydro-1alpha,3beta,3abeta-trihydroxy-2beta,10-dimethoxy-3,8alpha,11abeta,11cbeta-tetramethyl-	Others	1.22	0.00	100.91	up

Ingenol	Others	1.23	0.00	0.28	down
Sedoheptulose	Sugars	1.20	0.00	0.49	down
4H-1-Benzopyran-4-one, 2-(3,5-dihydroxyphenyl)-3,5,7-trihydroxy-	Others	1.23	0.01	6.81	up
(1S,2S,6S,7S,9R,14S,16S,17R)-14,16-dihydroxy-4,15-dimethoxy-2,6,14,17-tetramethyl-10-oxatetracyclo[7.7.1.02,7.013,17]heptadec-4-ene-3,11-dione	Others	1.18	0.00	12.28	up
Pyrethrosin	Others	1.19	0.03	3.96	up
Daphnetoxin	Others	1.24	0.00	0.36	down
Santin	Flavonoids	1.23	0.01	2.83	up
Gedunin	Others	1.23	0.00	9.11	up
alpha-Hederin	Others	1.20	0.03	0.04	down
2,3',4,6-Tetrahydroxybenzophenone	Flavonoids	1.22	0.03	5.96	up
coproporphyrinogen III	Others	1.23	0.00	2.26	up
Canthiumine	Organic acids	1.23	0.00	3.07	up
1-(3,4-Dimethoxyphenyl)-1,2-ethanediol 1-O-b-D-glucoside	Others	1.06	0.01	4.02	up
N-Acetyldopamine	Others	1.23	0.00	0.47	down
Val-trp	Amino acids	1.23	0.00	0.28	down
Farrerol	Flavonoids	1.24	0.00	2.73	up
CID 431000	Others	1.23	0.00	0.46	down
alpha-Amanitine	Others	1.12	0.00	5.57	up
Corilagin	Others	1.03	0.01	5.80	up
3-[(2R,3R,4R,5S)-3,4-dihydroxy-5-(hydroxymethyl)oxolan-2-yl]oxy-2-(3,4-dihydroxyphenyl)-5,7-dihydroxychromen-4-one	Flavonoids	1.21	0.00	0.28	down

Carboxytolbutamide	Benzenoids	1.22	0.00	3.37	up
Aquifoliunine EIV	Alkaloids	1.22	0.01	0.27	down
Aquifoliunine E-III	Alkaloids	1.02	0.00	0.10	down
Cassiaside B2	Others	1.23	0.00	8.80	up
ADP-glucose	Nucleotides	1.21	0.00	0.01	down
Trigallic Acid	Others	1.21	0.00	2.13	up
5alpha-Androstane-3alpha,17beta-diol disulfate	Others	1.18	0.00	0.24	down
p-Coumaraldehyde	Others	1.24	0.00	3.51	up
3H-isoindol-1-yl (3S,4S,5R)-3,4,5-trihydroxy-5-(hydroxymethyl)tetrahydrofuran-2-carboxylate	Alkaloids	1.11	0.02	2.26	up
Rosmarinic acid	Others	1.22	0.00	2.04	up
Kaempferol-7-rhamnoside	Flavonoids	1.11	0.00	4.15	up
Glyceric acid 1,3-biphosphate	Organic acids	1.20	0.00	0.23	down
Lycoricidine	Alkaloids	1.24	0.00	0.23	down
[6]-Gingerdiol 3,5-diacetate	Others	1.21	0.02	0.38	down
Floionolic acid	Lipids	1.10	0.01	0.46	down
Diisodityrosine	Amino acids	1.22	0.00	0.06	down
Phenylalanyltyrosine	Amino acids	1.20	0.03	2.59	up
24-Acetyl-25-cinnamoylvulgaroside	Benzenoids	1.11	0.02	0.33	down
Palmidin B	Others	1.20	0.00	0.05	down
Palmidin A	Others	1.19	0.02	0.35	down
Diesaconitine	Alkaloids	1.16	0.00	5.97	up
beta-Cotonefuran	Others	1.23	0.00	0.09	down
17R-hydroxy-4Z,7Z,10Z,13Z,15E,19Z-docosahexaenoic acid	Lipids	1.23	0.00	0.50	down
Rhapontin	Others	1.22	0.00	0.05	down

(S)-Mevalonic acid-5-pyrophosphate	Organic acids	1.12	0.01	0.37	down
Rutamarin	Others	1.24	0.00	7.06	up
Guanosine-5'-diphosphate	Nucleotides	1.21	0.00	2.90	up
Met-Asp	Amino acids	1.21	0.00	2.83	up
4-O-beta-D-glucosyl-4-coumaric acid	Organic acids	1.11	0.00	0.17	down
2-Methyl-d-erythritol 2,4-cyclodiphosphate	Sugars	1.17	0.00	10.03	up
D-inositol-4-phosphate	Others	1.21	0.03	0.25	down
Glycyl-tyrosyl-tyrosyl-prolyl-threonine	Amino acids	1.24	0.00	0.07	down
5,6,7,8-Tetrahydrosarcinapterin	Others	1.15	0.00	0.21	down
6''-O-Acetylglycitin	Flavonoids	1.21	0.00	0.31	down
1-(2-Hydroxyphenyl)-3-(4-hydroxyphenyl)-2-propen-1-one	Others	1.19	0.03	3.69	up
H-Trp-ser-OH	Amino acids	1.22	0.00	0.34	down
Uridine-5'-diphosphate-glucose	Nucleotides	1.23	0.00	0.40	down
Pimpinellin	Others	1.21	0.00	0.37	down
Deserpidine	Alkaloids	1.11	0.01	7.94	up
1,7-Bis(4-hydroxyphenyl)-3-heptanone	Others	1.20	0.01	2.93	up
Narceinone	Others	1.06	0.01	3.56	up
Ethyl tetradecanoate	Others	1.21	0.00	0.23	down
3,5-Di-O-galloyl-4-O-digalloylquinic acid	Others	1.22	0.00	26.08	up
1-Naphthylacetylspermine	Benzenoids	1.19	0.02	0.32	down
3-tert-Butyl-4-hydroxyanisole	Others	1.23	0.00	0.43	down
Gentisate aldehyde	Others	1.24	0.00	0.21	down
Pradimicin A	Benzenoids	1.23	0.00	0.22	down
Myricanol 5-(6-galloylglucoside)	Benzenoids	1.24	0.00	0.39	down
gamma-Glutamylglutamate	Amino acids	1.23	0.01	0.37	down

Maleic acid	Organic acids	1.23	0.00	0.35	down
Carnosifloside I	Others	1.19	0.01	0.15	down
Phenol	Others	1.24	0.00	0.20	down
Isorhamnetin 3-O-alpha-rhamnopyranosyl-(1-2)-beta-galactopyranoside	Flavonoids	1.23	0.00	2.61	up
Salidroside	Others	1.24	0.00	2.93	up
Afzelechin-(4alpha->8)-afzelechin	Flavonoids	1.22	0.00	0.47	down
Ile-Thr-Tyr-Asp	Amino acids	1.24	0.00	5.51	up
Ile-Asp-Lys-Lys	Amino acids	1.17	0.03	0.11	down
Glu-Gln-Phe-Arg	Amino acids	1.24	0.00	24.27	up
Asp-Phe-Val-Glu	Amino acids	1.23	0.00	4.20	up
Val-His-Leu-Asp	Amino acids	1.22	0.01	2.71	up
Asp-Glu-His-Glu	Amino acids	1.23	0.00	0.26	down
Arg-Ala-Leu-Lys	Amino acids	1.22	0.04	10.37	up
Ala-Lys-Asn-Glu	Amino acids	1.23	0.00	2.04	up
Gal1-3GalNAc1-3Gal1-4Gal1-4GlcSp	Sugars	1.18	0.00	0.32	down
Tyr-Lys-Arg-Glu	Amino acids	1.09	0.02	2.03	up
Ser-Ile-Phe-Glu	Amino acids	1.19	0.04	2.43	up
1,2-Dimyristoyl-sn-glycero-3-phospho-l-serine	Others	1.23	0.00	0.04	down
4-Nitrophenyl phosphate	Others	1.22	0.00	0.24	down
4-[(5-Methyl-1h-Pyrazol-3-Yl)amino]-2-Phenylphthalazin-1(2h)-One	Others	1.23	0.00	0.41	down
1,2-Docosahexanoyl-sn-glycero-3-phosphocholine	Others	1.23	0.00	0.36	down
Leu-Glu-Phe-Glu	Amino acids	1.19	0.02	2.18	up
Tyr-Glu-Arg-Arg	Amino acids	1.23	0.00	4.11	up

TyrMe-Trp-OH	Amino acids	1.23	0.00	0.24	down
Asn-Arg-Gln-Lys	Amino acids	1.24	0.01	10.35	up
Galb1-4GlcNAcb1-3Galb1-4Glcb-Sp	Sugars	1.13	0.01	2.40	up
Gal1-3[Fuc1-4]GlcNAcSp	Sugars	1.17	0.01	0.44	down
kaempferol 3-O-beta-D-glucopyranosyl-7-O-alpha-L-rhamnopyranoside	Flavonoids	1.18	0.01	0.12	down
Trp-HoPhe-OH	Amino acids	1.21	0.00	0.18	down
Ser-Val-Asn-Glu	Amino acids	1.23	0.00	4.57	up
Tyr-Nap-OH	Amino acids	1.23	0.00	0.09	down
Laricitrin 3-glucoside	Flavonoids	1.17	0.00	0.03	down
Ala-His-Leu-Asp	Amino acids	1.22	0.00	3.75	up
Nap-Asp-OH	Amino acids	1.22	0.00	0.21	down
TyrMe-Ser-OH	Amino acids	1.22	0.01	0.31	down
Asp-Gln-Ala-Asp	Amino acids	1.21	0.00	0.19	down
n6-(cis-hydroxyisopentenyl)adenosine	Nucleotides	1.14	0.05	3.33	up
Nap-His-OH	Amino acids	1.23	0.00	0.42	down
Ile-Ala-Leu-Arg	Amino acids	1.24	0.00	47.96	up
L-Asparagine, N-[2-(acetylamino)-2-deoxy-beta-D-glucopyranosyl]-	Amino acids	1.21	0.00	0.35	down
TyrMe-Asn-OH	Amino acids	1.24	0.00	0.27	down
Phe-Val-Ile-Glu	Amino acids	1.23	0.01	0.35	down
His-Gln-Val-Lys	Amino acids	1.22	0.01	12.98	up
Ile-Asp-Leu-Arg	Amino acids	1.24	0.00	31.38	up
2-Amino-5-benzoylbenzimidazole	Benzenoids	1.23	0.00	0.12	down
Nap-TyrMe-OH	Amino acids	1.24	0.00	2.35	up

2-[(1R,5S,6R,11R,13R,16S)-6-(furan-3-yl)-11-hydroxy-1,5,15,15-tetramethyl-8,14,17-trioxo-7-oxatetracyclo[11.3.1.02,11.05,10]heptadec-9-en-16-yl]acetic acid	Benzenoids	1.15	0.01	2.91	up
Quercetin 3-O-(6''-acetyl-glucoside)	Flavonoids	1.06	0.03	5.08	up
14-Methyl-3,12-dioxahexacyclo[16.3.1.04,21.05,10.011,20.015,19]docosa-5(10),7-diene-2,6,9-trione	Benzenoids	1.19	0.02	0.49	down
Phe-Nap-OH	Amino acids	1.22	0.01	0.48	down
[5-(5-Benzoyloxy-3,6,7-trimethoxy-4-oxochromen-2-yl)-2-methoxyphenyl] benzoate	Benzenoids	1.24	0.00	0.17	down
Mannose 6-phosphate	Sugars	1.22	0.01	0.37	down
Verbaspinoside	Benzenoids	1.23	0.00	0.39	down
N-Acetyl-DL-tryptophan	Amino acids	1.21	0.00	3.68	up
LPI(18:3/0:0)	Others	1.20	0.02	0.42	down
4-Coumaryl-4-coumarate	Others	1.23	0.00	0.44	down
KaempferoL-4-glucoside	Others	1.11	0.03	0.40	down
PG(16:1(9Z)/22:6(4Z,7Z,10Z,13Z,16Z,19Z))	Others	1.20	0.03	0.30	down
Syringetin-3-O-rutinoside-7-O-glucoside	Flavonoids	1.24	0.00	0.14	down
Hebevinoside I	Others	1.21	0.01	0.20	down
(5-{8-[3,7-dihydroxy-2-(3-hydroxyphenyl)-3,4-dihydro-2H-1-benzopyran-4-yl]-3,5,7-trihydroxy-3,4-dihydro-2H-1-benzopyran-2-yl}-2-hydroxyphenyl)oxidanesulfonic acid	Organic acids	1.22	0.00	6.44	up
8-[3,7-dihydroxy-2-(3-hydroxyphenyl)-3,4-dihydro-2H-1-benzopyran-4-yl]-6-[2-(3,4-dihydroxyphenyl)-3,7-	Benzenoids	1.16	0.00	2.14	up

dihydroxy-3,4-dihydro-2H-1-benzopyran-4-yl]-2-(3-hydroxyphenyl)-3,4-dihydro-2H-1-benzopyran-3,5,7-triol					
6-[3,7-dihydroxy-2-(3-hydroxyphenyl)-3,4-dihydro-2H-1-benzopyran-4-yl]-8-[3,7-dihydroxy-2-(4-hydroxy-3-methoxyphenyl)-3,4-dihydro-2H-1-benzopyran-4-yl]-2-(3,4-dihydroxyphenyl)-3,4-dihydro-2H-1-benzopyran-3,5,7-triol	Benzenoids	1.23	0.00	3.00	up
(4-ethenyl-2,6-dihydroxyphenyl)oxidanesulfonic acid	Organic acids	1.23	0.00	2.00	up
schisandrin C	Others	1.23	0.00	2.43	up
[5,6-Dihydroxy-2-(hydroxymethyl)-4-(3,4,5-trihydroxybenzoyl)oxyoxan-3-yl] 3,4,5-trihydroxybenzoate	Benzenoids	1.22	0.00	2.61	up
6-(2-{3-[2,4-dihydroxy-3-(3-methylbut-2-en-1-yl)benzoyl]-4-(2,4-dihydroxyphenyl)-6-methyl-7-oxabicyclo[4.1.0]heptan-2-yl}-5-[(E)-2-(2,4-dihydroxyphenyl)ethenyl]-3-hydroxyphenoxy)-3,4,5-trihydroxyoxane-2-carboxylic acid	Organic acids	1.15	0.02	0.05	down
6-[3,7-dihydroxy-2-(3-hydroxyphenyl)-3,4-dihydro-2H-1-benzopyran-4-yl]-2-(3,4-dihydroxyphenyl)-8-[2-(3,4-dihydroxyphenyl)-3,7-dihydroxy-3,4-dihydro-2H-1-benzopyran-4-yl]-3,4-dihydro-2H-1-benzopyran-3,5,7-triol	Benzenoids	1.14	0.01	0.49	down
6-Geranylchrysin	Flavonoids	1.22	0.02	0.34	down
5,7-dihydroxy-2-(4-hydroxy-3-methoxyphenyl)-3,6-bis[3,4,5-trihydroxy-6-(hydroxymethyl)oxan-2-yl]-4H-chromen-4-one	Others	1.23	0.00	0.12	down
Phe-Ser-Tyr-Asp	Amino acids	1.18	0.04	0.30	down
[(6-{5,7-dihydroxy-4-oxo-2-phenyl-8-[3,4,5-trihydroxy-6-(hydroxymethyl)oxan-2-yl]-3,4-dihydro-2H-1-benzopyran-6-yl}-3,4,5-trihydroxyoxan-2-yl)methoxy]sulfonic acid	Organic acids	1.21	0.00	9.81	up

2-amino-4-({1-[(carboxymethyl)-C-hydroxycarbonimidoyl]-2-{[2,3-dihydroxy-1-(4-methoxyphenyl)propyl]sulfanyl}ethyl}-C-hydroxycarbonimidoyl)butanoic acid	Organic acids	1.23	0.01	0.18	down
Sesaminol 2-O-triglucoside	Benzenoids	1.17	0.01	0.19	down
Madasiatic acid	Others	1.23	0.00	3.02	up
LPE(18:3/0:0)	Others	1.21	0.02	0.44	down
Hexahydroxydiphenic acid	Organic acids	1.06	0.00	7.12	up
5,6,7,8-Tetrahydroxy-2-(3,4,5-trihydroxyphenyl)chromen-4-one	Others	1.21	0.00	4.23	up
[3-(3,5,6,7-tetrahydroxy-4-oxo-4H-chromen-2-yl)phenyl]oxidanesulfonic acid	Organic acids	1.22	0.00	0.47	down
[4-[(E)-2-[3,5-dihydroxy-4-[(E)-3-methylbut-1-enyl]phenyl]ethenyl]phenyl] hydrogen sulate	Organic acids	1.23	0.01	3.57	up
8-[3,7-dihydroxy-2-(3-hydroxyphenyl)-3,4-dihydro-2H-1-benzopyran-4-yl]-6-[3,7-dihydroxy-2-(4-hydroxy-3-methoxyphenyl)-3,4-dihydro-2H-1-benzopyran-4-yl]-2-(3,4-dihydroxyphenyl)-3,4-dihydro-2H-1-benzopyran-3,5,7-triol	Benzenoids	1.13	0.01	2.09	up
[2,5-Dihydroxy-6-(hydroxymethyl)-3-(3,4,5-trihydroxybenzoyl)oxyoxan-4-yl] 3,4,5-trihydroxybenzoate	Benzenoids	1.23	0.02	5.00	up
2-(4-Hydroxyphenyl)-3-(3,5-dihydroxyphenyl)-4-(4-hydroxystyryl)benzofuran-6-ol	Benzenoids	1.13	0.00	17.41	up
palmyramide A	Lipids	1.01	0.02	0.13	down
(1S,4aS,7aS)-7-methyl-1-[(2S,3R,4S,5S,6R)-3,4,5-trihydroxy-6-(hydroxymethyl)tetrahydropyran-2-yl]oxy-1,4a,5,7a-tetrahydrocyclopenta[c]pyran-4-carboxylic acid	Others	1.21	0.00	0.35	down

Indole-3-ylacetyl-myo-inositol	Alkaloids	1.23	0.00	0.28	down
6-(2-ethyl-5-hydroxy-4-methoxyphenoxy)-3,4,5-trihydroxyoxane-2-carboxylic acid	Organic acids	1.20	0.00	0.23	down
2-Sinapoyloxy-3-butenylglucosinolate	Others	1.23	0.00	0.42	down
5-Phosphonooxy-L-lysine	Amino acids	1.20	0.05	0.32	down
2-amino-4-({1-[(carboxymethyl)-C-hydroxycarbonimidoyl]-2-{[2-hydroxy-1-(4-methoxyphenyl)-3-oxopropyl]sulfanyl}ethyl}-C-hydroxycarbonimidoyl)butanoic acid	Organic acids	1.19	0.00	0.49	down
6,11-dihydroxy-2,2,5-trimethyl-12-(6,8,11-trihydroxy-2,2,5-trimethyl-10-oxo-3,4,5,10-tetrahydro-2H-1-oxa-5-azatetraphen-4-yl)-5,10-dihydro-2H-1-oxa-5-azatetraphen-10-one	Others	1.23	0.00	0.12	down
Swertianolin	Flavonoids	1.24	0.00	7.36	up
3-(3,4-dimethoxyphenyl)-N-{2-[3-methoxy-4-(sulfooxy)phenyl]ethyl}prop-2-enimidic acid	Benzenoids	1.24	0.00	6.60	up
3,4,5-trihydroxy-6-[2-hydroxy-4-(3,5,7-trihydroxy-6-methoxy-4-oxo-3,4-dihydro-2H-1-benzopyran-2-yl)phenoxy]oxane-2-carboxylic acid	Organic acids	1.21	0.00	6.77	up
8-[3,4,5-trihydroxy-6-(hydroxymethyl)oxan-2-yl]-2-(2,4,5-trihydroxyphenyl)-3,4-dihydro-2H-1-benzopyran-3,5,7-triol	Benzenoids	1.23	0.00	0.47	down
Flavanone	Flavonoids	1.21	0.00	0.30	down
NCGC00381206-01_C40H60O10_1-Naphthalenebutanoic acid, decahydro-beta,6-dihydroxy-alpha-(2-hydroxyethylidene)-5-(hydroxymethyl)-5,8a-dimethyl-2-methylene-, (4E)-4-[2-[(1R,4aS,5R,6R,8aS)-decahydro-6-	Others	1.20	0.00	0.41	down

hydroxy-5-(hydroxymethyl)-5,8a-dimethyl-2-methylene-1-naphthalenyl]ethylidene]tetrahydro-5-oxo-3-furanyl ester, (alphaE,1R,4aS,5R,6R,8aS)-					
4-Hydroxypropranolol	Benzenoids	1.19	0.00	0.33	down
2-Dodecylbenzenesulfonic acid	Benzenoids	1.22	0.00	4.29	up
Ncgc00380975-01_C24H26O10_	Benzenoids	1.05	0.02	0.47	down
3-[5,7-dihydroxy-2-(4-hydroxy-3-methoxyphenyl)-4-oxo-3,4-dihydro-2H-1-benzopyran-8-yl]-5,7-dihydroxy-2-(4-hydroxyphenyl)-3,4-dihydro-2H-1-benzopyran-4-one	Benzenoids	1.18	0.00	0.30	down
3-[4-(2-Hydroxyethyl)piperazin-1-ium-1-yl]propane-1-sulfonate	Organic acids	1.09	0.04	0.46	down
6,8-bis[3,7-dihydroxy-2-(3-hydroxyphenyl)-3,4-dihydro-2H-1-benzopyran-4-yl]-2-(3,4-dihydroxyphenyl)-3,4-dihydro-2H-1-benzopyran-3,5,7-triol	Benzenoids	1.23	0.00	0.37	down
2-(3,4-dihydroxyphenyl)-5,6,7-trihydroxy-3,4-dihydro-2H-1-benzopyran-3-yl 3,4,5-trihydroxybenzoate	Benzenoids	1.20	0.00	0.07	down
[3-(3,5,7-Trihydroxy-6-methoxy-4-oxochromen-2-yl)phenyl] hydrogen sulate	Organic acids	1.23	0.00	2.22	up
{[4-(6,11-dihydroxy-2,2,5-trimethyl-10-oxo-5,10-dihydro-2H-1-oxa-5-azatetraphen-12-yl)-6,11-dihydroxy-2,5-dimethyl-10-oxo-3,4,5,10-tetrahydro-2H-1-oxa-5-azatetraphen-2-yl]methoxy}sulfonic acid	Organic acids	1.21	0.00	3.20	up
3-[5,7-dihydroxy-2-(3-hydroxyphenyl)-4-oxo-3,4-dihydro-2H-1-benzopyran-8-yl]-5,7-dihydroxy-2-phenyl-3,4-dihydro-2H-1-benzopyran-4-one	Benzenoids	1.22	0.01	2.82	up
{4-[(2E)-3-phenylprop-2-enoyl]phenyl}oxidanesulfonic acid	Organic acids	1.20	0.00	0.14	down

3-(3,4-dimethoxyphenyl)-N-{2-[4-methoxy-3-(sulfooxy)phenyl]ethyl}propanimidic acid	Organic acids	1.21	0.00	2.98	up
3-(3,4-dimethoxyphenyl)-N-[2-(3,4-dimethoxyphenyl)ethyl]-3-(sulfooxy)propanimidic acid	Benzenoids	1.23	0.00	0.46	down
Phe-Pro-Lys	Amino acids	1.21	0.02	2.58	up
6-[4-[(E)-2-(3,5-dihydroxyphenyl)ethenyl]phenoxy]-3,4,5-trihydroxyoxane-2-carboxylic acid	Organic acids	1.24	0.00	0.34	down
5,7-dihydroxy-2-(2,4,5-trihydroxyphenyl)-3,4-dihydro-2H-1-benzopyran-3-yl 3,4,5-trihydroxybenzoate	Benzenoids	1.23	0.00	0.31	down
6-[3,4-dihydroxy-2-(3-methylbut-2-en-1-yl)phenoxy]-3,4,5-trihydroxyoxane-2-carboxylic acid	Organic acids	1.21	0.00	11.35	up
1-O-glucosinoyl-β-D-glucose	Others	1.12	0.02	3.78	up
6-[3,4,5-trihydroxy-6-(hydroxymethyl)oxan-2-yl]-2-(2,4,5-trihydroxyphenyl)-3,4-dihydro-2H-1-benzopyran-3,5,7-triol	Benzenoids	1.23	0.00	0.35	down
5,7-dihydroxy-8-[3,4,5-trihydroxy-6-(hydroxymethyl)oxan-2-yl]-2-(2,4,5-trihydroxyphenyl)-4H-chromen-4-one	Others	1.01	0.04	2.99	up
8-[3,7-dihydroxy-2-(3-hydroxyphenyl)-3,4-dihydro-2H-1-benzopyran-4-yl]-6-[2-(3,4-dihydroxyphenyl)-3,7-dihydroxy-3,4-dihydro-2H-1-benzopyran-4-yl]-2-(4-hydroxy-3-methoxyphenyl)-3,4-dihydro-2H-1-benzopyran-3,5,7-triol	Benzenoids	1.07	0.00	0.25	down
3,4-dihydroxy-5-[3-hydroxy-4-methoxy-5-(3,4,5-trihydroxybenzoyloxy)benzoyloxy]benzoic acid	Organic acids	1.19	0.00	0.43	down
6-Methylthioguanosine monophosphate	Nucleotides	1.24	0.00	2.83	up
5,6,7-trihydroxy-2-(4-hydroxyphenyl)-8-(3,4,5-trihydroxyoxan-2-yl)-4H-chromen-4-one	Others	1.24	0.00	7.86	up

4-(7-Methoxy-2-oxochromen-6-yl)butan-2-yl hydrogen sulate	Organic acids	1.01	0.00	5.47	up
Asn-Glu-Glu	Amino acids	1.13	0.01	0.48	down
5-(3,4-Diacetoxybut-1-ynyl)-2,2'-bithiophene	Others	1.18	0.02	2.43	up
Arg-Ile-Val	Amino acids	1.23	0.00	0.27	down
[4-(2-oxo-3-phenylpropyl)phenyl]oxidanesulfonic acid	Organic acids	1.24	0.00	0.26	down
CID 74977437	Others	1.21	0.00	0.30	down
LysoPE(0:0/16:0)	Others	1.24	0.00	6.46	up
[2-(acetyloxy)-2-[6-(2-methylbut-3-en-2-yl)-7-oxo-2H,3H,7H-furo[3,2-g]chromen-2-yl]propoxy]sulfonic acid	Organic acids	1.24	0.00	0.25	down
Glutathionate	Amino acids	1.24	0.00	0.38	down
(R)-3-(indol-3-yl)-2-oxobutyric acid	Alkaloids	1.14	0.00	0.13	down
2-[2-(3,7-dimethylocta-2,6-dien-1-yl)-5-hydroxy-3-methoxyphenyl]-1-benzofuran-4,6-diol	Others	1.22	0.00	2.23	up
6-{3-[3-(3,5-dihydroxyphenyl)-2-oxopropanoyl]-2,4,6-trihydroxyphenoxy}-3,4,5-trihydroxyoxane-2-carboxylic acid	Organic acids	1.22	0.00	8.02	up
2-{3-[3-(2,4-dihydroxyphenyl)propanoyl]-2,6-dihydroxyphenyl}-4-methyl-6-(2,4,5-trihydroxyphenyl)cyclohex-3-ene-1-carboxylic acid	Organic acids	1.22	0.02	0.25	down
6-{2-[(E)-2-(3,5-dihydroxyphenyl)ethenyl]-5-{[3,4,5-trihydroxy-6-(hydroxymethyl)oxan-2-yl]oxy}phenoxy}-3,4,5-trihydroxyoxane-2-carboxylic acid	Organic acids	1.23	0.00	0.20	down
1-[3-(3,7-dimethylocta-2,6-dien-1-yl)-2,4,6-trihydroxyphenyl]-3-(3-hydroxyphenyl)propan-1-one	Others	1.24	0.00	4.25	up

{8-[2-(acetyloxy)propan-2-yl]-2-oxo-2H,8H,9H-furo[2,3-h]chromen-9-yl}oxidanesulfonic acid	Organic acids	1.24	0.00	0.20	down
5,7-dihydroxy-2-(3-hydroxyphenyl)-6-[3,4,5-trihydroxy-6-(hydroxymethyl)oxan-2-yl]-8-(3,4,5-trihydroxyoxan-2-yl)-4H-chromen-4-one	Others	1.21	0.00	0.43	down
Tyr-Abu-OH	Amino acids	1.22	0.03	0.14	down
2,3-dimethoxy-5-[7-methoxy-3-methyl-5-(prop-1-en-1-yl)-2,3-dihydro-1-benzofuran-2-yl]phenol	Others	1.21	0.00	0.31	down
3,4,5-trihydroxy-6-{[3,4,7-trihydroxy-2-(4-hydroxyphenyl)-3,4-dihydro-2H-1-benzopyran-5-yl]oxy}oxane-2-carboxylic acid	Organic acids	1.09	0.03	6.10	up
(2S,3S,4S,5R)-3,4,5-Trihydroxy-6-[2-hydroxy-5-(3,5,7-trihydroxy-3,4-dihydro-2H-chromen-2-yl)phenoxy]oxane-2-carboxylic acid	Organic acids	1.23	0.00	0.33	down
3,4,5-trihydroxy-6-{[2-(3-hydroxyphenyl)-5-methoxy-6,8-dimethyl-4-oxo-3,4-dihydro-2H-1-benzopyran-7-yl]oxy}oxane-2-carboxylic acid	Organic acids	1.21	0.00	2.26	up
6-({3,5-dihydroxy-2-[3-hydroxy-4-(methoxymethyl)phenyl]-3,4-dihydro-2H-1-benzopyran-7-yl}oxy)-3,4,5-trihydroxyoxane-2-carboxylic acid	Organic acids	1.24	0.00	2.70	up
Trp-Cys	Amino acids	1.24	0.00	0.17	down
3,4,5-trihydroxy-6-[4-(3,4,5,7-tetrahydroxy-3,4-dihydro-2H-1-benzopyran-2-yl)phenoxy]oxane-2-carboxylic acid	Organic acids	1.20	0.01	5.44	up
3'-Hydroxyflavanone	Others	1.23	0.00	5.10	up
His-Asn-Phe-Lys	Amino acids	1.22	0.01	0.22	down

6-[5-({[3,5-dihydroxy-6-(hydroxymethyl)-4-(3,4,5-trihydroxybenzoyloxy)oxan-2-yl]oxy}carbonyl)-2,3-dihydroxyphenoxy]-3,4,5-trihydroxyoxane-2-carboxylic acid	Organic acids	1.20	0.02	2.50	up
8-[2-(acetyloxy)-1-hydroxypropan-2-yl]-2-oxo-2H,8H,9H-furo[2,3-h]chromen-9-yl(2E)-2-methylbut-2-enoate	Others	1.21	0.00	0.38	down
3,4,5-Trihydroxy-6-[4-(3-oxo-3-{2,3,4-trihydroxy-5-[hydroxy(3,4,5-trihydroxyoxan-2-yl)methyl]-6-oxo-3-[3,4,5-trihydroxy-6-(hydroxymethyl)oxan-2-yl]cyclohexa-1,4-dien-1-yl}propyl)phenoxy]oxane-2-carboxylic acid	Benzenoids	1.23	0.00	3.91	up
Met-HoPhe-OH	Others	1.21	0.04	3.94	up
9,10-Dihydroxystearic acid	Lipids	1.22	0.01	0.42	down
3,4,5-trihydroxy-6-{2,4,6-trihydroxy-3-[3-(4-methoxyphenyl)propanoyl]phenoxy}oxane-2-carboxylic acid	Organic acids	1.19	0.04	0.21	down
CID 13988312	Others	1.14	0.00	4.78	up
2-{2,6-dihydroxy-4-[6-hydroxy-7-(3-methylbut-2-en-1-yl)-1-benzofuran-2-yl]phenyl}-6-(2,4-dihydroxyphenyl)-4-methylcyclohex-3-ene-1-carboxylic acid	Organic acids	1.22	0.00	3.35	up
6-[4-({[3,5-dihydroxy-6-(hydroxymethyl)-4-(3,4,5-trihydroxybenzoyloxy)oxan-2-yl]oxy}carbonyl)-2,6-dihydroxyphenoxy]-3,4,5-trihydroxyoxane-2-carboxylic acid	Organic acids	1.10	0.01	2.99	up
6-[[10-Butanoyl-3-hydroxy-6-(2-hydroxypropyl)-2,2-dimethyl-8-oxo-3,4-dihydropyrano[3,2-g]chromen-5-yl]oxy]-3,4,5-trihydroxyoxane-2-carboxylic acid	Organic acids	1.22	0.01	0.35	down
Epigallocatechin 3,5-digallate	Flavonoids	1.23	0.01	0.24	down

1-hydroxy-2-[6-(2-methylbut-3-en-2-yl)-7-oxo-2H,3H,7H-furo[3,2-g]chromen-2-yl]propan-2-yl acetate	Others	1.22	0.00	2.19	up
2-O-Acetylpseudolycorine	Others	1.23	0.00	0.16	down
6-{5-[5,7-dihydroxy-3-(3,4,5-trihydroxybenzoyloxy)-3,4-dihydro-2H-1-benzopyran-2-yl]-2-hydroxyphenoxy}-3,4,5-trihydroxyoxane-2-carboxylic acid	Organic acids	1.19	0.01	0.31	down
6-{2-[3-(3,5-dihydroxyphenyl)-2-oxopropanoyl]-3,4,5-trihydroxyphenoxy}-3,4,5-trihydroxyoxane-2-carboxylic acid	Organic acids	1.19	0.01	5.51	up
TyrMe-His-OH	Amino acids	1.23	0.00	0.08	down
2-O-alpha-L-Rhamnopyranosyl-D-glucopyranose	Sugars	1.24	0.00	0.49	down
2-amino-4-({1-[(carboxymethyl)-C-hydroxycarbonimidoyl]-2-{[1-hydroxy-1-(4-methoxyphenyl)-3-oxopropan-2-yl]sulfanyl}ethyl}-C-hydroxycarbonimidoyl)butanoic acid	Organic acids	1.23	0.01	0.16	down
(2S,3S,4S,5R)-6-[[3,5-Dihydroxy-2-(4-hydroxy-3-methoxyphenyl)-3,4-dihydro-2H-chromen-7-yl]oxy]-3,4,5-trihydroxyoxane-2-carboxylic acid	Organic acids	1.23	0.00	0.41	down
6-{[(16S)-5,7-dihydroxy-8,8,10,16-tetramethyl-3-[1-(2-methyl-1,3-thiazol-4-yl)prop-1-en-2-yl]-12-methylidene-9-oxo-17-oxa-4-azabicyclo[14.1.0]heptadec-4-en-11-yl]oxy}-3,4,5-trihydroxyoxane-2-carboxylic acid	Organic acids	1.09	0.00	8.07	up
6-[6-(3,4-Dihydroxy-6-methyl-5-oxooxan-2-yl)-7-hydroxy-2-(4-hydroxy-3-methoxyphenyl)-4-oxochromen-5-yl]oxy-3,4,5-trihydroxyoxane-2-carboxylic acid	Organic acids	1.23	0.00	4.95	up

3,4,5-trihydroxy-6-{[8-(2-hydroxypropan-2-yl)-4-(3-hydroxypropyl)-2-oxo-2H,8H,9H-furo[2,3-h]chromen-5-yl]oxy}oxane-2-carboxylic acid	Organic acids	1.21	0.00	5.29	up
[2,2-Dimethyl-6-(3,5,7-trihydroxy-4-oxochromen-2-yl)-3,4-dihydrochromen-3-yl] hydrogen sulate	Organic acids	1.22	0.02	0.23	down
Asn-Val-Gln-Asp	Amino acids	1.10	0.01	5.09	up
2-(3-hydroxyphenyl)-6-[3,4,5-trihydroxy-6-(hydroxymethyl)oxan-2-yl]-3,4-dihydro-2H-1-benzopyran-3,5,7-triol	Benzenoids	1.20	0.00	0.31	down
2-(3,4-dihydroxyphenyl)-8-[3,4,5-trihydroxy-6-(hydroxymethyl)oxan-2-yl]-3,4-dihydro-2H-1-benzopyran-3,5,6,7-tetrol	Benzenoids	1.21	0.00	0.08	down
PE(18:1(11Z)/20:2(11Z,14Z))	Others	1.18	0.03	0.21	down
oxalicine B	Others	1.22	0.00	0.21	down
(3-Ethoxyphenyl)-(2,3,4-trihydroxyphenyl)methanone	Benzenoids	1.23	0.01	0.26	down
Glu-Tyr-Asp-Lys	Amino acids	1.22	0.00	3.79	up
12-(6,11-dihydroxy-2,2,5-trimethyl-10-oxo-3,4,5,10-tetrahydro-2H-1-oxa-5-azatetraphen-4-yl)-6,8,11-trihydroxy-2,2,5-trimethyl-5,10-dihydro-2H-1-oxa-5-azatetraphen-10-one	Others	1.23	0.01	19.68	up
{4-[2,3-dioxo-3-(2,4,6-trihydroxy-3-methoxyphenyl)propyl]-2-methoxyphenyl}oxidanesulfonic acid	Organic acids	1.13	0.03	0.43	down
3,4,5-trihydroxy-6-{[(16S)-5,7,11-trihydroxy-8,8,10,12-tetramethyl-3-[1-(2-methyl-1,3-thiazol-4-yl)prop-1-en-2-yl]-9-oxo-17-oxa-4-azabicyclo[14.1.0]heptadec-4-en-16-yl]methoxy}oxane-2-carboxylic acid	Organic acids	1.23	0.00	3.93	up

1-Palmitoyl-2-(5-keto-6-octendioyl)-sn-glycero-3-phosphatidylcholine	Others	1.20	0.00	0.31	down
Thr-Asn-Phe-Asp	Amino acids	1.17	0.01	2.01	up
Shanciol A	Others	1.17	0.00	9.88	up
Trp-TyrMe-OH	Amino acids	1.11	0.03	0.44	down
Soyasaponin gamma-a	Others	1.08	0.02	0.32	down
6-{4-[2,3-dioxo-3-(2,4,6-trihydroxyphenyl)propyl]-2-hydroxyphenoxy}-3,4,5-trihydroxyoxane-2-carboxylic acid	Organic acids	1.18	0.01	0.13	down
2-(3-hydroxyphenyl)-1-(2,4,6-trihydroxyphenyl)propan-1-one	Others	1.15	0.02	2.18	up
6-[(3-Carboxy-3-hydroxy-2-{[3-(4-hydroxy-3-methoxyphenyl)prop-2-enoyl]oxy}propanoyl)oxy]-3,4,5-trihydroxyoxane-2-carboxylic acid	Benzenoids	1.23	0.01	9.59	up
1-Palmitoyl-2-(5-keto-8-oxo-6-octenoyl)-sn-glycero-3-phosphatidylcholine	Others	1.19	0.01	0.13	down
3-[(1R,2S,3S,5Z,7S,8S,9Z,13S,14Z,17R,18R)-3,13,17-tris(2-carboxyethyl)-2,7,18-tris(carboxymethyl)-1,2,5,7,12,12,15,17-octamethyl-3,8,13,18,19,22-hexahydrocorrin-8-yl]propanoic acid	Organic acids	1.18	0.00	0.11	down
6-{[2,2-dimethyl-6-(3-oxo-3-phenylprop-1-en-1-yl)-2H-chromen-5-yl]oxy}-3,4,5-trihydroxyoxane-2-carboxylic acid	Benzenoids	1.24	0.00	0.02	down
2-amino-4-({1-[(carboxymethyl)-C-hydroxycarbonimidoyl]-2-[(1-hydroxy-2,4-diphenylbutan-2-yl)sulfanyl]ethyl}-C-hydroxycarbonimidoyl)butanoic acid	Organic acids	1.15	0.01	2.09	up
Lys-Leu-Arg-Asp	Amino acids	1.19	0.04	0.19	down

(5-{5,7-dihydroxy-3-[(3,4,5-trihydroxyoxan-2-yl)oxy]-5H-chromen-2-yl}-2-hydroxyphenyl)oxidanesulfonic acid	Organic acids	1.22	0.02	2.70	up
1-Palmitoyl-2-(5-hydroxy-8-oxo-6-octenedioyl)-sn-glycero-3-phosphatidylcholine	Others	1.13	0.05	0.45	down
11-Hydroxyiridodial glucoside pentaacetate	Others	1.13	0.00	4.54	up
6-{2-[3-(3,7-dimethylocta-2,6-dien-1-yl)-7-hydroxy-4-oxo-4H-chromen-2-yl]-5-hydroxyphenoxy}-3,4,5-trihydroxyoxane-2-carboxylic acid	Organic acids	1.20	0.01	0.48	down
2-(2,4-dihydroxy-3-methoxyphenyl)-3,5,7-trihydroxy-5H-chromen-5-yl	Others	1.23	0.00	4.59	up
bracteatin 6-O-beta-glucoside	Benzenoids	1.21	0.03	6.52	up
Leu-Met-OH	Amino acids	1.19	0.00	3.02	up
UDP-L-arabinofuranose	Nucleotides	1.13	0.01	0.36	down
Naringenin-7-O--D-Glucuronide	Flavonoids	1.19	0.00	3.00	up
19-Hydroxytabersonine	Alkaloids	1.12	0.02	0.50	down
Thr-Tyr-Ile-Arg	Amino acids	1.16	0.00	0.33	down
dTDP-3-acetamido-3,6-dideoxy-alpha-D-galactopyranose	Nucleotides	1.24	0.00	0.10	down
Trunkamide	Others	1.15	0.03	0.35	down
3,4,5-trihydroxy-6-{[3-(3,4,5-trimethoxyphenyl)prop-2-enoyl]oxy}oxane-2-carboxylic acid	Benzenoids	1.21	0.03	0.32	down
Naringenin-4'-o-beta-d-glucuronide	Flavonoids	1.24	0.00	3.25	up
2-amino-4-({1-[(carboxymethyl)-C-hydroxycarbonimidoyl]-2-{[1-(3,4-dimethoxyphenyl)-2-{[2-(3,4-dimethoxyphenyl)ethyl]-C-hydroxycarbonimidoyl}-2-hydroxyethyl]sulfanyl}ethyl}-C-hydroxycarbonimidoyl)butanoic acid	Organic acids	1.22	0.00	0.30	down

6-{[3,5-dihydroxy-2-(3-methoxyphenyl)-3,4-dihydro-2H-1-benzopyran-7-yl]oxy}-3,4,5-trihydroxyoxane-2-carboxylic acid	Organic acids	1.06	0.02	0.31	down
PE(16:0/22:5(4Z,7Z,10Z,13Z,16Z))	Others	1.19	0.00	3.34	up
6-{[3,5-dihydroxy-2-(3-hydroxyphenyl)-8-[3,5,7-trihydroxy-2-(3-hydroxyphenyl)-6-[3,4,5-trihydroxy-6-(hydroxymethyl)oxan-2-yl]-3,4-dihydro-2H-1-benzopyran-4-yl]-3,4-dihydro-2H-1-benzopyran-7-yl]oxy}-3,4,5-trihydroxyoxane-2-carboxylic acid	Organic acids	1.05	0.03	0.08	down
Cys-Pro-Lys	Amino acids	1.19	0.01	4.04	up
Nap-Tyr-OH	Amino acids	1.22	0.03	0.10	down
3-[4-hydroxy-3-(3-methylbut-2-en-1-yl)phenyl]-1-(2,4,6-trihydroxyphenyl)propan-1-one	Others	1.24	0.00	22.12	up
PE(16:1(9Z)/22:6(4Z,7Z,10Z,13Z,16Z,19Z))	Others	1.23	0.00	3.01	up
Sericetin diacetate	Others	1.23	0.00	2.05	up
2-amino-4-({1-[(carboxymethyl)-C-hydroxycarbonimidoyl]-2-{[1-(6,7-dimethoxy-2H-1,3-benzodioxol-5-yl)-1,3-dihydroxypropan-2-yl]sulfanyl}ethyl}-C-hydroxycarbonimidoyl)butanoic acid	Organic acids	1.22	0.02	0.02	down
Met-Ser-Lys	Amino acids	1.18	0.03	4.68	up
7-HYDROXY-7-DESACETOXYKHIVORINIC ACID,METHYL ESTER	Others	1.23	0.00	0.43	down
6-[1-carboxy-2-(4-methoxy-1-benzofuran-5-yl)-2-oxoethyl]-3,4,5-trihydroxyoxane-2-carboxylic acid	Organic acids	1.22	0.00	27.26	up

2-(2,4-dihydroxy-5-methoxyphenyl)-8-[3,4,5-trihydroxy-6-(hydroxymethyl)oxan-2-yl]-3,4-dihydro-2H-1-benzopyran-3,5,7-triol	Benzenoids	1.22	0.01	0.08	down
6-({2-[4-ethyl-3-(sulfooxy)phenyl]-3,5-dihydroxy-3,4-dihydro-2H-1-benzopyran-7-yl}oxy)-3,4,5-trihydroxyoxane-2-carboxylic acid	Organic acids	1.22	0.00	3.42	up
6-[2-(acetyloxy)-2-(9-{[(2E)-2-methylbut-2-enoyl]oxy}-2-oxo-2H,8H,9H-furo[2,3-h]chromen-8-yl)propoxy]-3,4,5-trihydroxyoxane-2-carboxylic acid	Organic acids	1.02	0.04	0.33	down
Trypanothione	Organic acids	1.19	0.00	0.48	down
[3,4-Dihydroxy-5-[4-hydroxy-4-(hydroxymethyl)-3-sulooxyoxolan-2-yl]oxy-6-[5-hydroxy-2-(4-hydroxyphenyl)-4-oxochromen-7-yl]oxyoxan-2-yl]methyl acetate	Organic acids	1.23	0.00	0.28	down
Tyr-Ser-Val-Glu	Amino acids	1.15	0.01	2.07	up
5,6-dihydroxy-1,1,4a,6-tetramethyl-5-{[(2-oxo-2H-chromen-7-yl)oxy]methyl}-decahydronaphthalen-2-yl acetate	Others	1.21	0.01	2.09	up
5-[(E)-2-(4-{6-[2,4-dihydroxy-3-(3-methylbut-2-en-1-yl)benzoyl]-5-(2,4-dihydroxyphenyl)-3-methylcyclohex-2-en-1-yl}-3,5-dihydroxyphenyl)ethenyl]benzene-1,2,4-triol	Benzenoids	1.21	0.00	0.42	down
[4-(3,5,7-trihydroxy-3,4-dihydro-2H-1-benzopyran-2-yl)-2-{[3,4,5-trihydroxy-6-(hydroxymethyl)oxan-2-yl]oxy}phenyl]oxidanesulfonic acid	Organic acids	1.10	0.00	0.22	down
Ile-Tyr	Amino acids	1.22	0.00	0.50	down
Naringin chalcone	Others	1.22	0.00	0.15	down

2-amino-4-({1-[(carboxymethyl)-C-hydroxycarbonimidoyl]-2-{[1-hydroxy-1-(4-hydroxy-3-methoxyphenyl)-3-oxobutan-2-yl]sulfanyl}ethyl}-C-hydroxycarbonimidoyl)butanoic acid	Organic acids	1.15	0.01	0.16	down
3,4,5-trihydroxy-6-[4-(5-hydroxy-7-methoxy-8-methyl-4-oxo-4H-chromen-3-yl)-2-methoxyphenoxy]oxane-2-carboxylic acid	Organic acids	1.23	0.00	5.81	up
Isorhamnetin-3-O-rhamnoside	Flavonoids	1.20	0.01	3.05	up
3-Dehydro-2-deoxyecdysone	Others	1.24	0.00	0.35	down
Ile-Asn	Amino acids	1.23	0.00	17.86	up
6-{[2-(3,4-dihydroxy-5-methoxyphenyl)-3,5-dihydroxy-5H-chromen-7-yl]oxy}-3,4,5-trihydroxyoxane-2-carboxylic acid	Organic acids	1.21	0.02	2.75	up
Ser-Gln-Asp-Glu	Amino acids	1.18	0.01	2.48	up
Flavonol 3-O-beta-D-glucosyl-(1->2)-beta-D-glucoside	Flavonoids	1.14	0.00	2.97	up
3,4,5-trihydroxy-6-{[7-hydroxy-2-(4-hydroxyphenyl)-4-oxo-3,6-bis[3,4,5-trihydroxy-6-(hydroxymethyl)oxan-2-yl]-4H-chromen-5-yl]oxy}oxane-2-carboxylic acid	Organic acids	1.09	0.02	0.49	down
(+)-catechin 7-O-beta-D-xyloside	Flavonoids	1.16	0.00	0.10	down
6-[(2-{[3-(3,4-dimethoxyphenyl)-7-methoxy-8-methyl-4-oxo-4H-chromen-5-yl]oxy}-3,5-dihydroxy-6-(hydroxymethyl)oxan-4-yl)oxy]-3,4,5-trihydroxyoxane-2-carboxylic acid	Organic acids	1.21	0.01	0.09	down
2-(4-hydroxy-3-methoxyphenyl)-8-[3,4,5-trihydroxy-6-(hydroxymethyl)oxan-2-yl]-3,4-dihydro-2H-1-benzopyran-3,5,6,7-tetrol	Benzenoids	1.24	0.00	0.08	down
Asp-Met-Tyr	Amino acids	1.21	0.00	0.29	down

{[1-hydroxy-1-(7-methoxy-2-oxo-2H-chromen-8-yl)-3-methylbut-3-en-2-yl]oxy}sulfonic acid	Organic acids	1.24	0.00	3.72	up
1,3,6-Tri(4-hydroxybenzyl)-4-methoxy-9,10-dihydrophenanthrene-2,7-diol	Benzenoids	1.21	0.00	2.51	up
Lyciumoside III	Others	1.18	0.04	2.31	up
3,4,5-trihydroxy-6-({7-methoxy-4-oxo-2-phenyl-8-[3,4,5-trihydroxy-6-(hydroxymethyl)oxan-2-yl]-4H-chromen-5-yl}oxy)oxane-2-carboxylic acid	Organic acids	1.16	0.01	0.49	down
6-(2-{6-[2,4-dihydroxy-3-(3-methylbut-2-en-1-yl)benzoyl]-5-(2,4-dihydroxyphenyl)-4-hydroxy-3-methylcyclohex-2-en-1-yl}-5-[(E)-2-(2,4-dihydroxyphenyl)ethenyl]-3-hydroxyphenoxy)-3,4,5-trihydroxyoxane-2-carboxylic acid	Organic acids	1.20	0.00	0.16	down
3,4,5-trihydroxy-6-[2-methoxy-4-(3-oxobut-1-en-1-yl)phenoxy]oxane-2-carboxylic acid	Benzenoids	1.23	0.01	0.22	down
1-Vaccenoyl-2-myristoyl-sn-glycerol	Others	1.18	0.00	0.14	down
Curcumin diglucoside	Others	1.19	0.00	7.70	up
[3,4-dihydroxy-6-[5-hydroxy-2-(4-hydroxyphenyl)-4-oxo-7-[3,4,5-trihydroxy-6-(hydroxymethyl)oxan-2-yl]oxychromen-3-yl]oxy-5-[(2S,3R,4R,5R,6S)-3,4,5-trihydroxy-6-methyloxan-2-yl]oxyoxan-2-yl]methyl acetate	Benzenoids	1.09	0.03	0.11	down
3,4,5-trihydroxy-6-{4-[3-(5-hydroxy-2,2-dimethyl-2H-chromen-6-yl)prop-2-enoyl]phenoxy}oxane-2-carboxylic acid	Benzenoids	1.23	0.00	0.22	down
